# Parental rights or parental wrongs: Parents’ metacognitive knowledge of the factors that influence their school choice decisions

**DOI:** 10.1371/journal.pone.0301768

**Published:** 2024-04-18

**Authors:** Trent N. Cash, Daniel M. Oppenheimer

**Affiliations:** 1 Department of Social and Decision Sciences, Carnegie Mellon University, Pittsburgh, Pennsylvania, United States of America; 2 Department of Psychology, Carnegie Mellon University, Pittsburgh, Pennsylvania, United States of America; Alzahra University, IRAN (ISLAMIC REPUBLIC OF)

## Abstract

School choice initiatives–which empower parents to choose which schools their children attend–are built on the assumptions that parents know what features of a school are most important to their family and that they are capable of focusing on the most important features when they make their decisions. However, decades of psychological research suggest that decision makers lack metacognitive knowledge of the factors that influence their decisions. We sought to reconcile this discrepancy between the policy assumptions and the psychological research. To do so, we asked participants to complete Choice-Based Conjoint surveys in which they made series of choices between different hypothetical schools. We then asked participants to self-report the weight they placed on each attribute when making their choices. Across four studies, we found that participants did not know how much weight they had placed on various school attributes. Average correlations between stated and revealed weights ranged from *r* = .34–.54. Stated weights predicted different choices than revealed weights in 16.41–20.63% of decisions. These metacognitive limitations persisted regardless of whether the participants were parents or non-parents (Study 1a/1b), the nature of the attributes that participants used to evaluate alternatives (Study 2), and whether or not decision makers had access to school ratings that could be used as metacognitive aids (Study 3). In line with prior psychological research–and in contract to policy assumptions–these findings demonstrate that decision makers do not have particularly strong metacognitive knowledge of the factors that influence their school choice decisions. As a result, parents making school choice decisions are likely to seek out and use the wrong information, thus leading to suboptimal school choices. Future research should replicate these results in more ecologically valid samples and test new approaches to school choice that account for these metacognitive limitations.

## Introduction

### School choice in America

A recent Gallup poll found that only about a third of Americans (36%) were satisfied with the quality of the American K-12 educational system [1]. This is, unfortunately, hardly an anomaly: since 1999, satisfaction levels have ranged from a low of 36% (2000; 2023) to a high of 53% (2004). Given these persistently low satisfaction levels, politicians [2] and advocacy organizations [3] have proposed a variety of educational reforms, collectively known as school choice, in which parents evaluate a variety of schools and choose the one that best fits their family’s needs, rather than sending their child to their neighborhood public school [4–6]. One reason for which school choice advocates argue that these policies will lead to better outcomes is that they believe parents have the most accurate knowledge of what their children need in a school [7].

### School choice efficacy

To date, empirical scholarship regarding the efficacy of school choice has been mixed [8–12]. Recent reviews and meta-analyses of the extant literature have suggested that, on average, school choice policies have small, positive impacts on educational attainment [13], academic achievement [5], and disciplinary issues [10]. However, these positive effects have been found to be moderated by demographic characteristics [5] and highly variable based on the quality of available alternative schools [14]. Despite the heterogeneity of results, the literature clearly suggests that, although school choice policies may have some benefits, they have not been the panacea that proponents suggest they should be.

One theory to explain the limited efficacy of school choice is that its success is dependent on families having sufficient resources to make good decisions. This is perhaps best exemplified by studies demonstrating that, compared to high-SES families, low-SES families making school choice decisions limit their choice set to a smaller number of schools due to practical constraints, such as commute time [15, 16]. Many empirical accounts have also highlighted the fact that the complex nature of the school choice decision environment places a large burden on parents’ limited cognitive resources by providing too many options [7, 17], too much information [18], and information that is uninformative or difficult to understand [19, 20].

### Metacognitive knowledge of attribute weights

An additional explanation that has received little empirical attention is the hypothesis that parents may lack sufficient knowledge about what they value in a school and thus struggle to choose a school that matches their priorities [2, 3, 6]. Because school choice is a multi-attribute decision, parents’ priorities are expressed through an attribute weighting process in which they determine the relative weight they should place on each attribute on which schools can be compared [21]. For example, parents must ask themselves which is more important: the quality of the math curriculum, the graduation rate, or the commute time to get to the school. A decision maker’s ability to generate and apply these weights in a way that truly matches their priorities reflects their ability to make decisions based on the factors that matter most to them [22].

To successfully weight attributes in a way that matches their true preferences, decision makers must monitor their beliefs and values [23, 24] and control the application of these beliefs and values during the decision process [25, 26]. As such, attribute weighting can be considered a metacognitive process [27–30]. For this reason, we will refer to a decision maker’s awareness of the influence that various factors have (and ought to have) on their school choice decisions as their metacognitive knowledge of attribute weights.

### Benchmarking metacognitive knowledge of attribute weights

The extant literature has shown that decision makers often lack the metacognitive capacities to accurately and consistently self-report the reasons for which they make decisions [31]. Studies seeking to quantify this inconsistency have demonstrated that, on average, correlations between attribute weights elicited via different modalities (e.g., self-reported vs. revealed) typically fall within in the range of *r* = .40 - .70 [32–38]. The range is wide because existing studies have implemented different preference elicitation methods, evaluated different domains, and induced different psychological goal states [32–38]. While these studies were designed to test (in)consistencies across different preference elicitation methods–not necessarily metacognitive knowledge itself–this range of typical correlations provides a useful benchmark against which to which to compare participants’ metacognitive knowledge of attribute weights, particularly because our paradigm compares self-reported weights to revealed weights.

### Metacognitive knowledge of attribute weights in school choice

If the limited metacognitive ability demonstrated by decision makers in other domains holds true in school choice decisions, it would have implications for the success of school choice policies. If parents lack sufficient metacognitive knowledge of attribute weights, they may choose schools that do not actually align with their priorities. Similarly, parents who lack metacognitive knowledge of their educational priorities may be unable to accurately respond to polls or surveys about how they want local educational leaders to improve their schools [39–41]. For these reasons, it is imperative that we empirically investigate the degree to which parents have accurate metacognitive knowledge of their educational priorities.

Despite the evidence regarding decision makers’ lack of metacognitive knowledge in general [31, 36, 37], the high value that many individuals and communities place on education may make school choice a domain in which decision makers have a uniquely high level of metacognitive knowledge. School choice priorities are also highly influenced by cultural norms [42, 43] that may provide decision makers with easily accessible scripts about what ‘people like them’ value [44, 45], thus granting an avenue for decision makers to be metacognitively aware of the factors that influence their decisions. As such, metacognitive knowledge may be greater in school choice than other domains that have typically been studied.

### The current study

To date, however, empirical studies of metacognitive knowledge in the school choice domain are lacking. To rectify this, we conducted four experiments in which participants were tasked with making school choice decisions and then reporting on how heavily they weighted various attributes when making their decisions. In Studies 1a and 1b, we showed that participants were unable to accurately report the weight they placed on various attributes, regardless of whether they were a general online sample (1a) or a sample of parents of high school-aged children (1b). In Study 2, we replicated this finding using a different set of attributes, some of which were non-academic in nature. Finally, In Study 3, we further replicated this result while giving participants access to meta-cognitive aids in the form of aggregate school ratings that graded each alternative school on an A-F Scale. Across studies, our results suggest that metacognitive limitations should be considered as a potential roadblock to the success of school choice policies and should be accounted for in policy design.

All participants affirmed their written informed consent to participate at the beginning of each study and all procedures were conducted in compliance with and approved by the Carnegie Mellon University Institutional Review Board (IRB). Data, materials, and code for all studies reported in this manuscript are available at: https://osf.io/krxec/?view_only=8b829144f7e54518957d3334521c0774

## Study 1a

### Study 1a overview

Study 1a aimed to evaluate the accuracy of participants’ metacognitive knowledge of the weight they placed on various attributes when making school choice decisions. Participants completed a Choice Based Conjoint (CBC) survey in which they made a series of hypothetical choices among high schools for their children’s education based on a given set of attributes. Participants then reported the weight that they had placed on each attribute and how important each attribute was to them. Alignment between these measures was then used to evaluate participants’ metacognitive knowledge.

### Study 1a methods

#### Participants

An analysis of simulated respondents suggested that our Choice-Based Conjoint analysis would require approximately 200 responses to reduce the standard error of our utility estimates to less than 0.05 if they were estimated using logistic regression. This is the standard method of sample size estimation for CBC [46] and represents an upper-bound of the necessary sample size when using more precise utility estimation methods, such as Hierarchical Bayes, which we used. To ensure that our sample would be sufficiently large after removing participants who failed a bot check, we recruited 210 participants from MTurk via CloudResearch [47]. In this bot check, participants were asked to explain why a simple, pun-based cartoon was funny. Participants were marked as bots if they provided non-sense response or responses that were totally unrelated to the cartoon. Data was collected February 11^th^ - 16^th^, 2021. Nine participants were excluded for failing the bot check, leaving 201 participants. Due to a programming error, ten of these participants were unable to complete two portions of the study (details below), leaving a total of 191 participants (86 females, 161 White participants, 78 parents, *M*_age_ = 38.6 years) who completed the entire study. Full demographic characteristics are reported in the S1 Table.

#### CBC survey

Participants were asked to imagine that they were parents picking among high schools for their children to attend. They then completed a Choice Based Conjoint (CBC) survey in which they were tasked with picking between sets of three high schools with different scores on seven attributes. CBC is an established and well-validated tool from the marketing literature that researchers have used for decades to estimate the degree to which participants care about various attributes by having them repeatedly choose between alternatives that systematically vary across values of attributes [48–52]. CBC has been implemented in a wide variety of policy-relevant domains including medicine [53], transportation [54], nutrition [55], electoral politics [56], and many others. Discrete choice tasks like CBC have been used in the literature to assess school choice preferences [40], but have not been used to assess metacognitive knowledge of these preferences. The CBC survey was designed using Sawtooth Software Inc.’s Lighthouse Studio, V 9.15.4 [57].

#### Attribute selection

To ensure that our study included attributes that are normally accessible to parents, we first began by gathering all of the attributes used by three school rating websites: U.S. News & World Report [58], Niche [59], and GreatSchools [60]. We then reduced the set to only consider attributes that were given a weight of at least 10% by one of the school rating websites. We then selected attributes that we believed captured different aspects of school performance and would be easy for participants to understand and compare. In line with the literature on what attributes parents value in schools, we primarily focused on measures of academic performance and diversity [40].

The seven attributes that we ultimately decided to use were: 1) Graduation Rate; 2) Percent of Students Who Pass State Tests (henceforth, *State Test Pass Rate*); 3) The Gap in State Test Pass-Rates Between (Economically and Racially) Advantaged and Disadvantaged Students (*Disadvantaged Student Gap*); 4) Average ACT score; 5) Average Rating Given to the School by Parents (*Average Parent Rating*); 6) Percent of Seniors Who Take at Least One AP course (*AP Enrollment*); and 7) Percent of Students Who Are a Racial/Ethnic Minority (*Percent Minority Students*). Some of the other attributes that were used by the school rating websites that we chose not to use in our CBC included: Growth ratings, Number of AP courses offered, AP exam performance, and Extracurricular participation rates [58–60]. Most CBC studies include between 3–8 attributes, so having 7 attributes put us squarely in the standard range [61].

There were five possible levels for each attribute, which were selected to reflect the range of real schools’ performances on these metrics. In line with CBC conventions, we chose to have only five levels per attribute to reduce decision complexity for participants [61]. To ensure that participants understood the attributes, participants were provided with: 1) A brief overview of what the attribute measured; 2) an explanation of the five possible levels of the attribute; and 3) data regarding real world average scores on the attribute, which were based on publicly available state or national data. For complete attribute descriptions, see S1 Appendix.

#### CBC survey procedure

**Fig 1:**
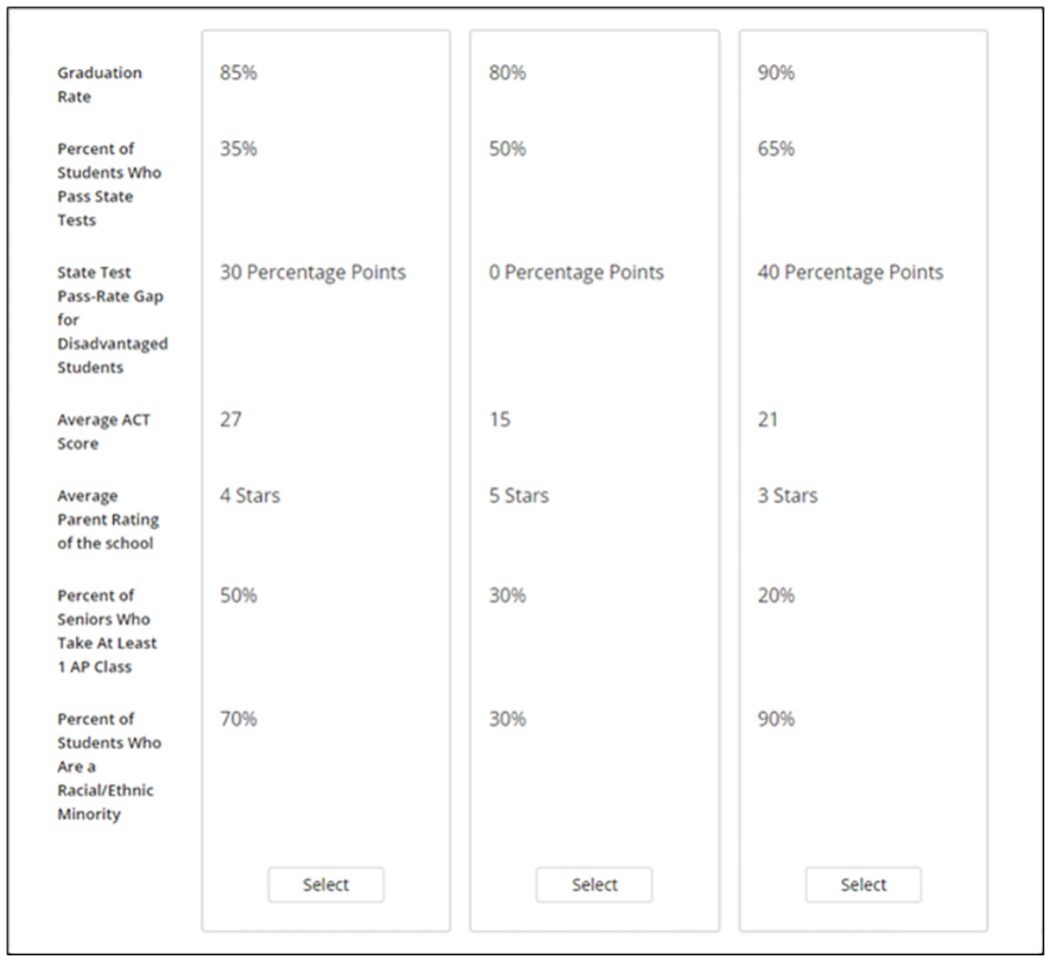
Sample CBC task from Study 1a.

During the CBC task, participants made 14 choices between sets of three hypothetical schools. This is typical of CBC studies, most of which ask participants to make 15 or fewer choices [61]. The attribute levels for the hypothetical schools were randomly generated for each participant. To avoid school options that seemed unrealistic, we prohibited the following attribute levels from co-occurring: 1) The lowest (highest) level of the State Test Pass Rate attribute (20% and 80%, respectively) could not be paired with the highest (lowest) level of the Average ACT Score attribute (27 and 15, respectively); and 2) The highest level of the AP Enrollment attribute (60%) could not be paired with the lowest level of the State Test Pass Rate attribute (20%) or the lowest level of the Average ACT Score attribute (15). The order of the seven attributes was held constant across participants. A sample task is depicted in Fig 1.

#### Self-reported weights procedure

After completing the CBC survey, participants were shown a list of the seven attributes and asked to rate how important each attribute was to them on a scale of 1 (“Not at All Important”) to 9 (“Extremely Important”). We refer to these ratings to as Attribute Importance Ratings (AIRs). Participants were then shown the list of attributes once more and asked to identify what percentage of their decisions had depended on the schools’ scores on each given attribute. We refer to these percentages as Stated Attribute Weights (SAWs). Mean SAWs and AIRs for each attribute are reported in the S2 Table. Finally, participants completed a brief demographic survey. Due to a programming error, the AIR and SAW items did not appear for 10 participants, leaving only 191 participants who completed all parts of the study.

### Study 1a analysis & results

#### Analytical decisions

The following decisions were made regarding how to conduct analyses for all studies presented in this manuscript. Outliers were not excluded. Participants with missing data were excluded in a listwise manner. A significance threshold of α = .05 was applied for all analyses. Analyses were conducted using Lighthouse Studio, V 9.15.4 [57] and R Statistical Software, V 4.3.2 [62]. Materials, data and analysis code for are available at: https://osf.io/krxec/?view_only=8b829144f7e54518957d3334521c0774

#### Estimating utilities

We first converted the CBC choice data into Revealed Attribute Weights (RAWs). To estimate these RAWs, we used Hierarchical Bayes Estimation (HB) to estimate utilities that captured the relative value that each participant placed on each level of each attribute (i.e., part-worth utilities) [49, 61, 63]. We began the HB estimation process with conservative priors of 0 for all part-worth utility parameters and updated the parameters over 20,000 iterations (10,000 to reach convergence, 10,000 retained and averaged for point estimates) using a Markov Chain Monte Carlo. Utility estimates for each iteration were generated using a Metropolis Hastings Algorithm (for a technical overview, see [64]). For ease of replicability, all settings were left at the default provided by Lighthouse Studio, V 9.15.4 [57].

We chose to use HB as our estimation method because it is considered the gold standard for estimating utilities from CBC data and because it allows for the generation of utility estimates at the individual-level, rather than the sample level [49, 61]. Additionally, HB uses sample-level utility estimates to inform individual-level estimates, thus generating more accurate utility estimates from relatively little data than older methods of assessing CBC data, like logistic regression [48–52, 61]. Our CBC survey adhered to standard conventions and parameters by having a relatively small number of attributes, choices, and levels [61]. As such, there is no reason to believe that HB would be an invalid or unreliable way to estimate utilities in the context of our study. We are further convinced of the reliability and validity of our HB estimation procedure because we achieve such similar results across the studies presented here.

#### Estimating RAWs from utilities

We then converted the individual utilities into RAWs, which are estimated as percentages and can be interpreted as the weight that each participant placed on each attribute. In adherence to standard conventions [61, 63], RAWs for each attribute for each participant were calculated according to the following equation, where *U*_*j*_ is a vector containing the utility values for the five levels of attribute *j*, *U*_*i*_ is a vector containing the utility values for the five levels of attribute *i*, and the set from which attribute *i* is pulled includes all seven attributes from the CBC, including *j*:

$$RAW_{j}=100*\;\frac{\text{max}\left(U_{j}\right)-\text{min}\left(U_{j}\right)}{\sum_{i=1}^{N}\left(\text{max}\left(U_{i}\right)-\text{min}\left(U_{i}\right)\right)}$$

The best and worst levels of each attribute were determined separately for each participant, thus allowing us to capture participants’ heterogeneous preferences. RAWs were calculated for all 201 participants, but all analyses were conducted based on the reduced sample of 191 participants. Mean RAWs for each attribute are reported in the S2 Table. Both the HB estimations and the RAW calculations were conducted using Sawtooth Software Inc.’s Lighthouse Studio, V 9.15.4 [57].

#### Estimating perfect metacognitive knowledge

We next sought to establish what would constitute perfect metacognitive knowledge of attribute weights. To estimate this upper bound, we conducted a simulation in which simulated respondents (*n* = 191) completed the same CBC survey as the human participants. Each simulated respondent was yoked to one human participant’s set of SAWs. For each choice task, the value of each alternative was calculated by multiplying the assigned SAW for each attribute by the level of that attribute (scored as 1–5) and summing these values across the 7 attributes. The simulated respondent then selected the alternative with the highest sum value. We then used HB to calculate the RAWs for the simulated respondents.

**Fig 2:**
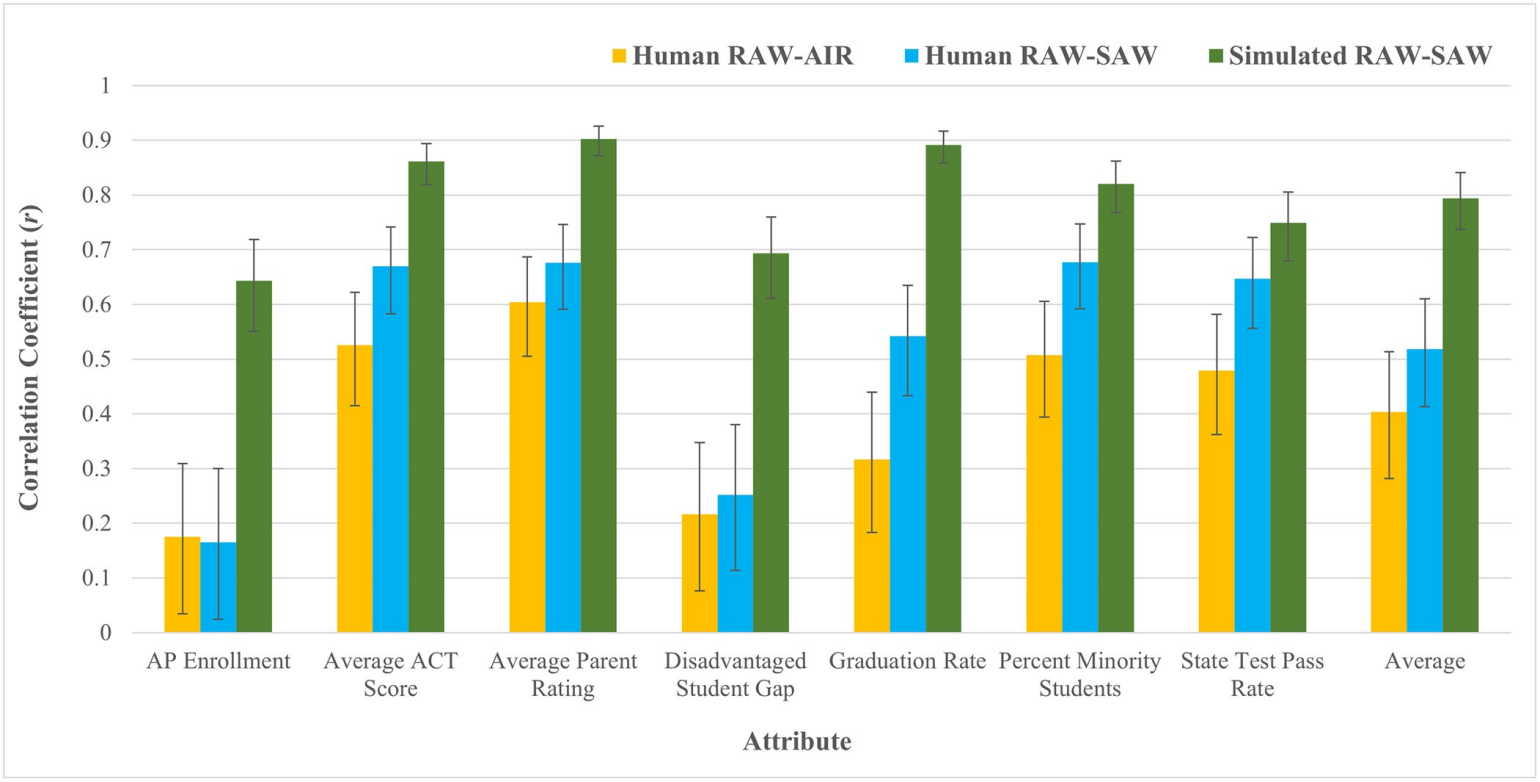
Study 1a RAW-AIR, RAW-SAW, and simulated RAW-SAW correlations, by attribute. Error bars reflect 95% confidence intervals.

The correlations between simulated respondents’ RAWs and assigned SAWs were then calculated for each attribute. These correlations ranged from *r* = .64 - .90 (see Fig 2) and the mean of the seven correlation coefficients was *r* = .79. Since simulated respondents’ assigned SAWs were yoked to human participants’ SAWs, these correlations approximated the correlations that participants would be expected to produce between RAWs and SAWs if they had perfect metacognitive knowledge of their attribute weights. These simulations reflect a fairer standard of comparison than perfection (*r* = 1.00), as some of participants’ miscalibration (i.e., the gap between *r* = .79 and *r* = 1.00) arises from the noise that is inherent to comparisons of weights generated via different measurement modalities. By running simulations instead of assuming perfection, we can determine if participants’ metacognitive knowledge is sub-optimal, even after removing the degree of miscalibration that is due to measurement error.

#### RAW-SAW correlations

To evaluate participants’ metacognitive knowledge of their attribute weights, we first evaluated the correlations between participants’ Revealed Attribute Weights (RAWs) and Stated Attribute Weights (SAWs) for each attribute. Correlations for each attribute ranged from *r* = .17 - .68 (see Fig 2), and the average of these seven correlation coefficients was only *r* = .52. This moderate correlation was typical of the degree of miscalibration found across preference elicitation modalities in the literature (*r* = .40 - .70) [37], suggesting that decision makers’ metacognitive knowledge of attribute weights is similarly limited in school choice as it is in other domains.

In order to test whether or not the correlations were significantly different from one another [65], we conducted a series of Fisher’s *r* to *z* transformations in which we compared the correlations between RAWs and assigned SAWs for the simulated respondents to RAW-SAW correlations achieved by the human participants for each of the seven attributes (See Fig 2). The human participants achieved significantly lower correlations than the simulated respondents for six of the seven attributes (*n*s = 191, *z*s = -7.95 –-3.24, *p*s ≤ .001). For *State Test Pass Rate*, the difference in correlations was marginally significant (*n*s = 191, *z* = -1.95, *p* = .05). The average of the human participants’ seven correlation coefficients (*r* = .52, *n* = 191) was significantly lower than the average of the simulated respondents’ seven correlation coefficients (*r* = .79, *n* = 191, *z* = -4.93, *p* < .001). These comparisons suggest that participants’ metacognitive knowledge of their own attribute weights was limited, even accounting for the noise that is inherent to comparisons across measurement modalities.

#### RAW-SAW different choice predictions

As a final test of the alignment between participants’ RAWs and SAWs, we estimated how often participants would have made different choices if they had based their decisions on their SAWs as opposed to their RAWs. To do so, we separately analyzed each choice task completed by each participant (*n* = 2,674 tasks) and estimated the utility that the participant would have assigned to each of the three schools by multiplying their SAW or RAW for each attribute by the level of the attribute (scored as 1–5) and summing across the seven attributes. We then assumed that the participant would select the alternative with the highest utility and calculated the percentage of tasks for which weighting by SAWs led to different choices than weighting by RAWs. We found that weighting by SAWs led to different choices than weighting by RAWs in 18.36% (491/2,674; κ = .72) of choice tasks.

#### RAW-AIR correlations

We also explored the correlations between participants’ RAWs and their attribute importance ratings (AIRs). Correlation coefficients for each attribute ranged from *r* = .18 - .60 (see Fig 2), and the average of the seven correlation coefficients was only *r* = .40. This low-to-moderate correlation falls at the bottom end of the benchmark range (*r* = .40 - .70) [37] for correlations between attribute weights measured via different elicitation methods, thus providing further evidence that decision makers lack metacognitive knowledge of the attribute weights they use when making school choice decisions, just as they do in other domains. A two-tailed fisher’s *r* to *z* transformation indicated that the mean correlation between RAWs and AIRs was not significantly different than the mean correlation between RAWs and SAWs (*n* = 191, *z* = -1.42, *p* = .16).

#### Demographic differences in metacognitive knowledge

We next explored possible demographic differences in metacognitive calibration. To do so, we first calculated the absolute difference between each participant’s RAW and SAW for each attribute, and then took the average of these values across attributes to generate a measure of each participant’s metacognitive knowledge of the attribute weights they had used. We then regressed this Average RAW-SAW Difference variable (descriptive statistics reported in the S2 Table) on six demographic factors: gender (male vs. female), parental status (parents vs. non-parents), educational attainment (Bachelor’s vs. no Bachelor’s), urban status (urban vs. suburban vs. rural), age, and income. Only the coefficient for income was significant (*B* = -0.38, *SE* = 0.15, *p* = .01). The full regression is reported in Table 1.

**Table 1:** Study 1a demographic factors predicting average RAW-SAW differences.

Dependent Variable: Average RAW-SAW Difference		Study 1a *R*^2^ = .06		
	*B*	*95% CI*	*SE*	*p*
Intercept	9.89***	7.27–12.51	1.33	< .001
Female	0.32	-0.69–1.34	0.51	.53
Age	<0.01	-0.04–0.05	0.02	.84
Suburban	-0.08	-1.23–1.07	0.58	.90
Rural	0.34	-1.09–1.77	0.72	.64
Non-Parent	-0.09	-1.17–0.99	0.55	.87
Bachelor’s Degree	-0.49	-1.50–0.53	0.51	.35
Income	-0.38*	-0.67 –-0.09	0.15	.01

Note.

*p < .05,

**p < .01,

***p < .001;

Male, Urban, Parent, and No Bachelor’s Degree were used as comparison groups; Similar regressions that used Rural as the comparison group indicated no significant effects of urban status.

### Study 1a discussion

In Study 1a, participants were unable to accurately self-report the weight that they had placed on various attributes when making school choice decisions. The degree of miscalibration was similar to results reported in the literature for other domains. This pattern persisted regardless of self-report format, and metacognitive knowledge was not associated with demographic characteristics other than income. In conjunction, these results suggest that decision makers may lack the metacognitive sophistication to make school choice decisions that match their true preferences. Indeed, we found that over 18% of choices would have been different if participants had applied the weighting functions that they claimed to have preferred, rather than the weighting functions they revealed through their choices.

Notably, however, the participants in Study 1a were a combination of parents and non-parents, which could mask the possibility that parents may have unique metacognitive knowledge of their school choice attribute weights because they are likely to have more experience with the education system. While Study 1a did find that parents and non-parents had statistically identical Average RAW-SAW Differences, the sample did not include enough parents to conduct the full set of analyses on parents alone. To rectify this limitation and fully address the possibility that parents could have different levels of metacognitive knowledge than non-parents, Study 1b replicated Study 1a with a participant sample consisting entirely of parents of high school-aged children.

## Study 1b

### Study 1b overview

Study 1b was conducted as a perfect replication of Study 1a, except that all participants were parents whose oldest children were high school-aged (14–19 years old) at the time of study completion. The purpose of Study 1b was to test whether parents of high school-aged children have greater metacognitive knowledge of attribute weights in school choice decisions than the general population. Participants completed the exact same tasks as in Study 1a.

### Study 1b methods

#### Participants

To ensure that our sample was sufficiently large, we recruited 225 participants from Prolific. Prolific was used instead of MTurk because MTurk did not have a filter for recruiting parents of high school-aged children. Data was collected May 30^th^–June 9^th^, 2023. 220 participants completed the study, but 18 were excluded because their children were not in the proper age range. We used the same bot check as in Study 1a. No participants failed the bot check. As such, we had a final sample of 202 parents (121 females, 167 White participants, Mean age of oldest child = 16.2 years, *M*_age_ = 44.9 years). Full demographic characteristics are reported in the S1 Table.

#### Procedures

All methodological and analytical procedures were identical to Study 1a.

### Study 1b results

#### Estimating revealed attribute weights

Revealed Attribute Weights (RAWs) were estimated from CBC responses using the same techniques as in Study 1a. Mean RAWs, SAWs, and AIRs for each attribute are reported in the S2 Table.

#### Estimating perfect metacognitive knowledge

**Fig 3:**
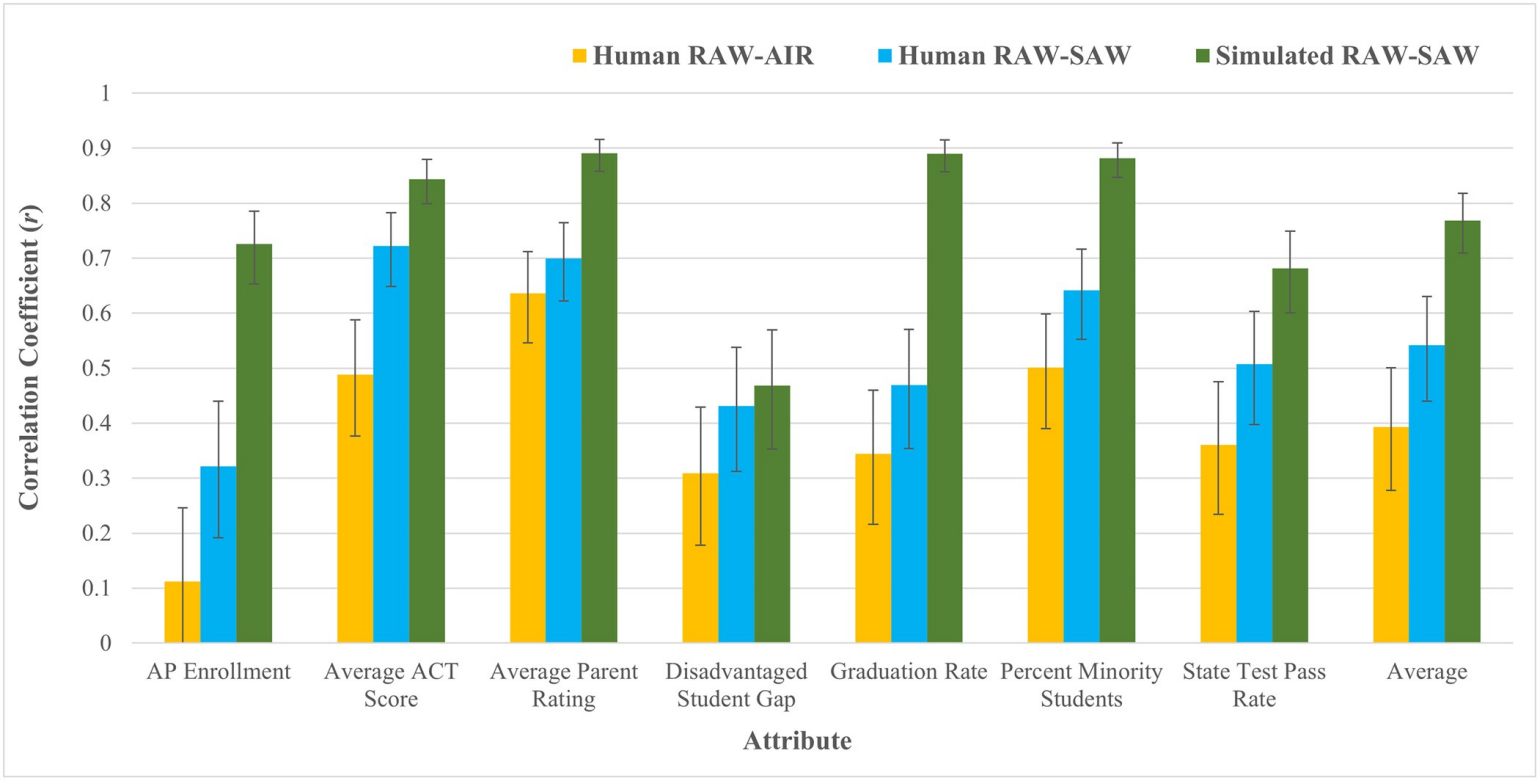
Study 1b RAW-AIR, RAW-SAW, and simulated RAW-SAW correlations, by attribute. Error bars reflect 95% confidence intervals.

As in Study 1a, we first ran simulated participants who completed the CBC survey using the human participants’ SAWs as their decision weights. The correlations between RAWs and assigned SAWs for the simulated respondents ranged from *r* = .47 - .89 (see Fig 3). The mean of the seven correlation coefficients was *r* = .77. Unsurprisingly, a two-tailed Fisher’s *r* to *z* test indicated that this average correlation was not significantly different than the average correlation for simulated participants from Study 1a (*r* = .79, *n*s = 202, *z* = -0.64, *p* = .52), suggesting that the benchmark of optimal metacognitive knowledge was identical for the two samples.

#### RAW-SAW correlations

We next evaluated the correlations between RAWs and SAWs for each attribute. Correlations for each attribute ranged from *r* = .32 - .72 (see Fig 3), and the average of these seven correlation coefficients was only *r* = .54 (*n* = 202). This moderate correlation falls in the middle of the benchmark range (*r* = .40 - .70) [37] for correlations between attribute weights measured via different elicitation methods, suggesting that metacognitive knowledge is similarly limited in school choice as it is in other domains. A two-tailed Fisher’s *r* to *z* transformation indicated that this average correlation was not significantly different than the average correlation from Study 1a (*r* = .52, *n* = 191, *z* = 0.32, *p* = .75), suggesting that the all-parent sample from Study 1b did not have greater or worse metacognitive knowledge of attribute weights than the mixed parent and non-parent sample from Study 1a.

We then compared the human participants’ correlations to the simulated participants’ correlations using two-tailed Fisher’s *r* to *z* transformations and found that, as in Study 1a, the human participants in Study 1b achieved significantly lower correlations than the simulated respondents for six of the seven attributes (*n*s = 202, *z*s = -9.08 –-2.73, *p*s = < .001–007; see Fig 3). For the *Disadvantaged Student Gap* attribute, the difference in correlations was not significant (*n*s = 202, *z* = -0.46, *p* = .65). Similarly, a two-tailed Fisher’s *r* to *z* transformation indicated that the average of the human participants’ seven correlation coefficients (*r* = .54, *n* = 202) was significantly lower than the average of the simulated respondents’ seven correlation coefficients (*r* = .77, *n* = 202, *z* = -4.09, *p* < .001), suggesting that the parents had sub-optimal metacognitive knowledge of attribute weights, even after accounting for the noise that is inherent to comparisons across measurement modalities.

#### RAW-SAW different choice predictions

Using the same procedures as Study 1a, we estimated the percentage of decisions (*n* = 2,828) for which participants would have made different choices if they used their SAWs as attribute weights rather than their RAWs. Weighting by SAWs led to different choices than weighting by RAWs in 16.41% (464/2,828; κ = .75) of choice tasks. A chi-square test for equality of proportions indicated that this was marginally (but not significantly) lower than the proportion in Study 1a, *X*^2^(1, *n*s = 191–202) = 3.53, *p* = .06, but it is nonetheless still a higher than desirable proportion, and again suggests that participants were making choices based on different attributes than they thought they were.

#### RAW-AIR correlations

We then evaluated correlations between RAWs and AIRs. Correlation coefficients for each attribute ranged from *r* = .11 - .64 (see Fig 3), and the average of the seven correlation coefficients was only *r* = .39. This weak correlation falls slightly below the benchmark range (*r* = .40 - .70) [37] for correlations between attribute weights measured via different elicitation methods, again highlighting the limitations of metacognitive knowledge of attribute weights in the school choice domain. A two-tailed Fisher’s *r* to *z* transformation indicated that this mean correlation was not significantly different from the mean correlation between RAWs and AIRs in Study 1a (*r* = .40, *n* = 191, *z* = -0.12, *p* = .90), suggesting similarly limited metacognitive knowledge for parents and the mixed parent/non-parent sample. A two-tailed Fisher’s *r* to *z* transformation also indicated that the mean correlation between RAWs and AIRs was marginally (but not significantly) different than the mean correlation between RAWs and SAWs (*r* = .54, *n* = 202, *z* = -1.91, *p* = .06). In isolation, this marginal effect is difficult to interpret, but in conjunction with the non-significant result from the comparable analysis in Study 1a (and later, Studies 2 and 3), provides some additional evidence that response format does not influence metacognitive knowledge of attribute weights. More importantly, however, both response formats suggest that participants’ metacognitive knowledge of the weight they place on various factors is limited.

#### Demographic differences in metacognitive knowledge

Finally, we regressed the same demographic characteristics as in Study 1a (except parental status, since all participants were parents; descriptive statistics reported in the S2 Table) on the Average RAW-SAW Difference variable. None of the coefficients were significant (*p*s = .22 - .70). This suggests that demographic characteristics do not significantly predict metacognitive knowledge of attribute weights. The full regression is reported in Table 2.

**Table 2:** Study 1b demographic factors predicting average RAW-SAW differences.

Dependent Variable: Average RAW-SAW Difference		Study 1b *R*^2^ = .02		
	*B*	*95% CI*	*SE*	*p*
Intercept	7.12***	4.07–10.16	1.54	< .001
Female	0.60	-0.36–1.57	0.49	.22
Age	0.02	-0.04–0.09	0.03	.52
Suburban	0.69	-0.41–1.78	0.55	.22
Rural	0.47	-0.97–1.91	0.73	.52
Bachelor’s Degree	0.22	-0.89–1.32	0.56	.70
Income	-0.08	-0.32–0.16	0.12	.50

Note.

*p < .05,

**p < .01,

***p < .001;

Male, Urban, and No Bachelor’s Degree were used as comparison groups; Similar regressions that used Rural as the comparison group indicated no significant effects of urban status. All participants in Study 1b were parents, so parental status was not included in the regression

### Study 1b discussion

In all, the results of Study 1b replicated the finding from Study 1a that decision makers were unable to accurately self-report the weight that they had placed on various attributes when making school choice decisions, thus reflecting a lack of metacognitive knowledge. This result persisted across demographics and regardless of response format. Study 1b did not replicate Study 1a’s finding that income was associated with Average RAW-SAW differences. Most importantly, Study 1b demonstrated that parents of high school-aged children do not have greater metacognitive knowledge of the attribute weights they use when making school choice decisions than a mixed sample of parents and non-parents. For this reason, all remaining studies use a mixed sample of parents and non-parents.

One limitation of Studies 1a and 1b is that they only included attributes that were highly weighted by school rating websites, which tend to prioritize academics. Despite academic factors’ importance in naturalistic contexts, placing such a high emphasis on them when evaluating metacognitive knowledge presents two challenges. First, academic factors are often correlated in the real world and may serve as substitutes for one another. Secondly, the emphasis on academic attributes may have driven participants who did not value academic factors highly to put less thought into their decisions, thus limiting their ability to display metacognitive knowledge. To rectify these concerns, Study 2 used the same methodology as Study 1a, but replaced some of the academic attributes with non-academic attributes that were rated as important by pilot subjects.

## Study 2

### Study 2 overview

Study 2 was conducted as a replication of Studies 1a, except that four of the academic-focused attributes from Study 1a were replaced with four non-academic attributes. The goal of Study 2 was to demonstrate that the findings from Study 1a and 1b were robust to a different attribute set. Participants completed the exact same tasks as in Studies 1a and 1b.

### Study 2 methods

#### Participants

To ensure that our sample would be sufficiently large after eliminating respondents who failed a bot check, we recruited 218 participants from MTurk via CloudResearch [47]. Data was collected April 8^th^–April 9^th^, 2021. Four participants were excluded for failing the same bot check that was used in Studies 1a and 1b, leaving 214 participants (95 females, 174 White participants, 90 parents, *M*_age_ = 39.1 years). Full demographic characteristics of these participants are reported in the S1 Table.

#### Procedures

Because this was a conceptual replication, Study 2 was nearly identical to Studies 1a and 1b, except that we replaced four of the school attributes with new attributes. To determine these attributes, we conducted two pilot studies (reported in the S2 Appendix) in which we asked participants to generate (Pilot Study 1) and rate the importance of (Pilot Study 2) attributes (*n* = 79) that parents care about when picking between schools. Pilot data was collected October 23^rd^–December 3^rd^, 2020. We then selected the four attributes that received the highest importance ratings that were: 1) not similar to any of the already-included attributes; 2) able to be operationalized into five objective levels (i.e., no dichotomous variables); 3) not a measure of academic performance; and 4) easy for a typical participant to understand. Based on these criteria, we selected: 1) Crime Rate per 1,000 students (henceforth, *Crime Rate*); 2) Average Teacher (State Licensing) Exam Score Percentile (*Teacher Exam Score*); 3) Per-Student Spending; and 4) Emotional Support Score (a measure of student ratings of emotional support).

In addition to these four new attributes, we retained three attributes that were weighted heavily by participants in Studies 1a and 1b: *Graduation Rate; State Test Pass Rate*; and *Average Parent Rating*. We opted not to include any prohibitions in Study 2, meaning that any level of any attribute could be paired with any level of any other attribute. To ensure that the effects were not driven by attribute order, the order in which attributes were presented was randomized at the participant level. All other procedures were identical to Studies 1a and 1b.

### Study 2 analysis & results

#### Estimating revealed attribute weights

Revealed Attribute Weights (RAWs) were estimated from CBC responses using the same techniques as in Studies 1a and 1b. Mean RAWs, SAWs, and AIRs for each attribute are reported in the S2 Table.

#### Estimating perfect metacognitive knowledge

**Fig 4:**
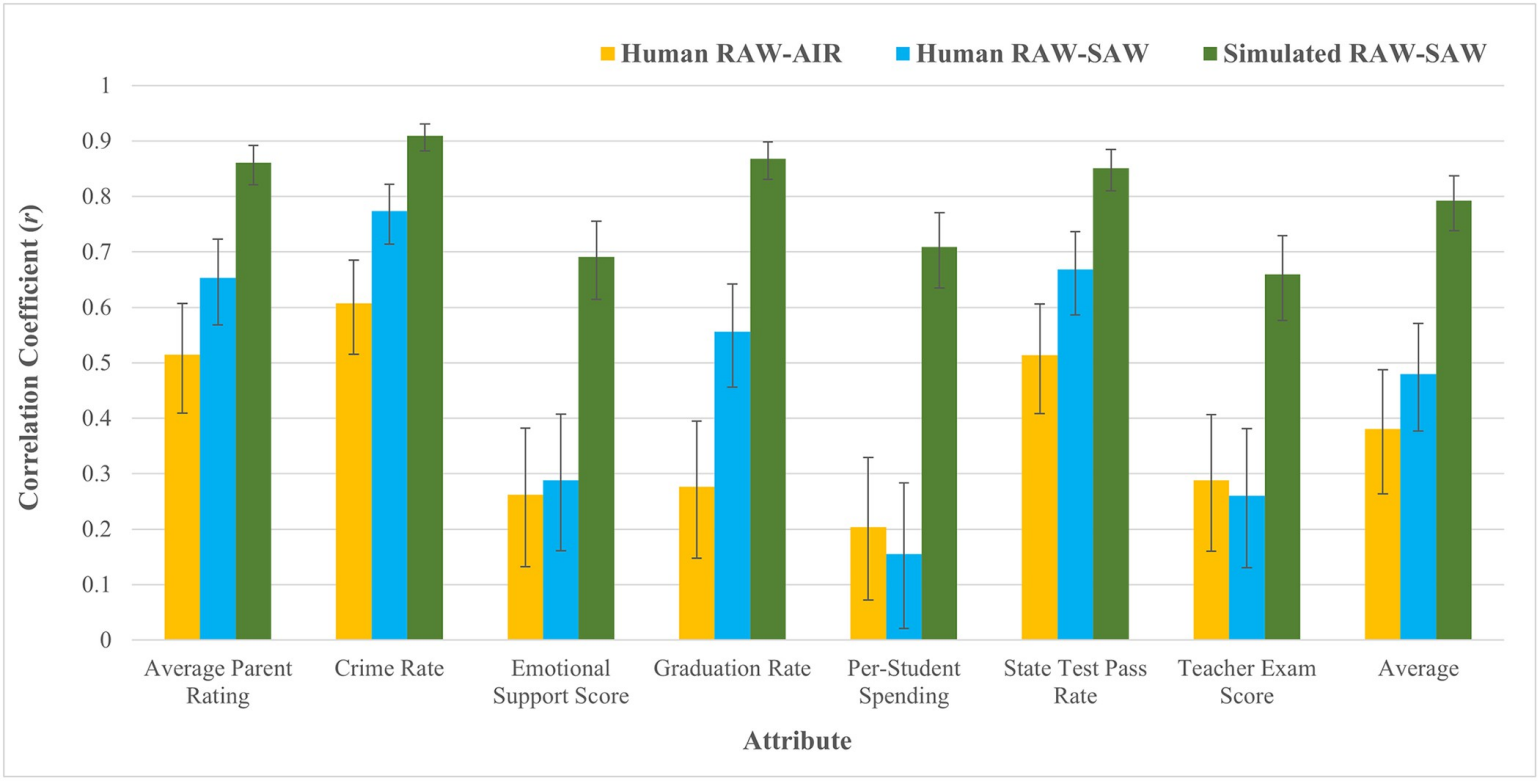
Study 2 RAW-AIR, RAW-SAW, and simulated RAW-SAW correlations, by attribute. Error bars reflect 95% confidence intervals.

As in Studies 1a and 1b, we estimated perfect metacognitive knowledge via simulated respondents (See Study 1a for details). The simulated correlations between assigned SAWs and estimated RAWs for each attribute ranged from *r* = .66 - .91 (see Fig 4), and the mean of these correlation coefficients was *r* = .79.

#### RAW-SAW correlations

We next evaluated the correlations between participants’ RAWs and SAWs for each attribute. Correlation coefficients for each attribute ranged from *r* = .15 - .77 (see Fig 4), and the mean of the seven correlation coefficients was *r* = .48. As in Studies 1a and 1b, this moderate correlation was typical of the degree of miscalibration across elicitation methods found in the literature (*r* = .40 - .70) [37]. Two-tailed Fisher’s *r* to *z* transformations indicated that the average correlation across attributes for Study 2 (*r* = .48, *n* = 214) was not significantly different than the average correlation across attributes for Study 1a (*r* = .52, *n* = 191; *z* = -0.52, *p* = .60) or Study 1b (*r* = .54, *n* = 202; *z* = -0.86, *p* = .39), suggesting that the lack of metacognitive knowledge of attribute weights identified in Studies 1a and 1b was not the result of an over-emphasis on academic attributes (at the expense of other, non-academic attributes).

We then used two-tailed Fisher’s *r* to *z* transformations to compare the correlations between RAWs and assigned SAWs for the simulated respondents to the correlations between RAWs and SAWs for the human participants. For all seven attributes, correlations for the human participants were significantly lower than correlations for the simulated respondents (*n*s = 214, *z*s = -7.49 –-4.68, *p*s < .001; see Fig 4). Similarly, a two-tailed Fisher’s *r* to *z* transformation indicated that the average of the human participants’ seven correlations (*r* = .48, *n* = 214) was lower than the average of the simulated respondents’ seven correlations (*r* = .79, *n* = 214, *z* = -5.72, *p* < .001). As in Studies 1a and 1b, these differences suggest that participants’ metacognitive knowledge was limited, even after accounting for the noise that is inherent to comparisons across measurement modalities.

#### RAW-SAW different choice predictions

Using the same procedures as Studies 1a and 1b, we estimated the percentage of decisions (*n* = 2,996) for which participants would have made different choices if they used their SAWs as attribute weights rather than their RAWs. We found that weighting by SAWs led to different choices than weighting by RAWs in 17.62% (528/2,996; κ = .74) of choice tasks. Chi-square tests of equality of proportions indicated that this proportion was not significantly different than the proportion from Study 1a, *X*^2^(1, *n*s = 191–214) = 0.47, *p* = .49, or Study 1b, *X*^2^(1, *n*s = 202–214) = 1.44, *p* = .23.

#### RAW-AIR correlations

We next evaluated correlations between RAWs and AIRs. Correlation coefficients for each attribute ranged from *r* = .20 - .61 (see Fig 4). The average of these correlation coefficients was only *r* = .38 (*n* = 214), which two-tailed Fisher’s *r* to *z* transformations indicated was not significantly different than the average RAW-AIR correlation from Study 1a (*r* = .40, *n* = 191, *z* = -0.27, *p* = .79) or Study 1b (*r* = .39, *n* = 202 *z* = -0.15, *p* = .88). This low-to-moderate correlation falls slightly below the benchmark range found in the literature for correlations between attribute weights measured via different elicitation methods (*r* = .40 - .70) [37], thus providing further evidence that decision makers’ metacognitive knowledge of attribute weights is limited in school choice decisions. A two-tailed fisher’s *r* to *z* transformation indicated that the mean correlation between RAWs and AIRs was not significantly different than the mean correlation between RAWs and SAWs (*r* = .48, *n* = 214, *z* = -1.24, *p* = .21).

#### Demographic differences in metacognitive knowledge

We next regressed participants’ Average RAW-SAW Differences (descriptive statistics reported in the S2 Table) on the same six demographic variables as in Studies 1a and 1b. None of the coefficients were significant (*p*s = .21 - .89), suggesting that the demographic variables did not effectively predict participants’ metacognitive knowledge. The full regression is reported in Table 3.

**Table 3:** Study 2 demographic factors predicting average RAW-SAW differences.

Dependent Variable: Average RAW-SAW Difference		Study 2 *R*^2^ = .03		
	*B*	*95% CI*	*SE*	*p*
Intercept	8.19***	5.76–10.63	1.23	< .001
Female	-0.07	-1.03–0.90	0.49	.89
Age	0.01	-0.04–0.06	0.02	.65
Suburban	0.73	-0.41–1.88	0.58	.21
Rural	0.20	-1.34–1.74	0.78	.80
Non-Parent	0.09	-0.99–1.17	0.55	.87
Bachelor’s Degree	-0.57	-1.63–0.48	0.53	.29
Income	-0.11	-0.36–0.14	0.13	.38

Note.

*p < .05,

**p < .01,

***p < .001;

Male, Urban, Parent, and No Bachelor’s Degree were used as comparison groups; Similar regressions that used Rural as the comparison group indicated no significant effects of urban status.

## Study 2 discussion

Study 2 provided a conceptual replication of Studies 1a and 1b. Most importantly, Study 2 demonstrated that the results of Studies 1a and 1b extend to a more diverse set of attributes than just academic attributes.

In the real world, however, parents have access to cognitive aids that they may use to improve their school choice decisions. The most common cognitive aids available to parents are school rating websites–such as U.S. News & World Report [58], Niche [59], and Greatschools [60]–that provide aggregate ratings of each school’s quality on a numeric or report card style (A-F) scale. Having access to these aggregate school ratings may help parents to better understand their own preferences and/or offload their decision making to experts in the field, thus improving decision makers’ metacognitive knowledge of the weight they place on various attributes (ostensibly parents would know whether they had followed the recommendation of an external rating system). To account for this possibility, Study 3 was designed as a conceptual replication of Studies 1a, 1b, and 2, but participants were given access to aggregate school ratings for each school, with varying levels of information about the aggregate school rating provided depending on condition.

## Study 3

### Study 3 overview

Study 3 was conducted as a conceptual replication of Studies 1a, 1b, and 2, except that participants in four experimental conditions were provided with an eighth attribute, *The Cash Report*, that served as an aggregate rating (on an A+-F scale) of the quality of a school. The purpose of Study 3 was to evaluate whether having access to a (meta-)cognitive aid like *The Cash Report* would improve participants’ metacognitive knowledge of the attribute weights they used in school choice decisions. Participants completed all the same tasks as in Studies 1a, 1b, and 2.

### Study 3 methods

#### Participants

To ensure that our sample would be sufficiently large after eliminating respondents who failed a bot check, we recruited 1,032 participants from MTurk via CloudResearch [47]. Data was collected July 22^nd^– 27^th^, 2021. Of the 1,032 participants we recruited, 34 (5–10 from each condition) were excluded for failing the same bot check that was used in the previous studies, leaving a total of 998 participants (483 females, 769 White participants, 475 parents, *M*_age_ = 39.4 years). Full demographic characteristics of these participants are reported in the S1 Table.

#### Experimental design

Participants were randomly assigned to one of five conditions. These conditions included a control condition (*n* = 197) that was identical to Study 1a/Study 1b and four experimental conditions (*n*s = 197–203) that were identical to either Study 1a/Study 1b or Study 2, except that participants were given an eighth attribute for each school called *The Cash Report*, which provided an aggregate rating for each school on an A+ to F scale. Participants in the experimental conditions were told that *The Cash Report* was a new school rating website that “*collects details about schools across the country and uses those details to rate each school on a report card-style A+ to F scale*” and that it was “*similar to other well-known school rating resources*, *such as U*.*S*. *News & World Report*, *Niche*, *and GreatSchools*.*com*.”

We opted to create a hypothetical school rating site rather than using one of the established sites to avoid potential confounds that may have arisen from participant familiarity with the existing sites. Given that we sought to manipulate knowledge of how the school ratings were calculated, it would have been a major confound if any participant was familiar with the formulas that the sites used to calculate their ratings. Using an existing site also would have introduced the possibility that some participants may have had positive or negative past experiences with the sites, which may have altered their usage of the school rating attribute in a way that was metacognitively accessible to them. The downside of using a hypothetical school rating site is that participants may have been less likely to give it significant weight because it was not a familiar brand, like the existing sites [58–60]. However, we do not consider this a critical limitation because a metacognitively calibrated participant should adjust for this factor in both their choices and their self-reports.

The four experimental conditions followed a standard 2x2 design in which we manipulated the following variables: 1) Which attribute set was presented alongside *The Cash Report* (Studies 1a/1b’s academic attribute set vs. Study 2’s mixed academic and non-academic attribute set); and 2) Whether or not participants were given information about the formula *The Cash Report* used to calculate its ratings (Known Formula vs. Unknown Formula). In the Known Formula conditions, the description of *The Cash Report* that was given to participants included a sentence stating that “*The Cash Report calculates its ratings primarily based on the following details for each school*:” followed by a list of the seven attributes from Study 1a/1b. No prohibitions were used in any condition. Attribute order was randomized in all conditions.

As a result of this 2x2 design, *The Cash Report* contained entirely redundant information for participants in the *Study 1 Known Formula* condition, but only partially redundant information (3/7 attributes) for participants in the *Study 2 Known Formula* condition. This distinction is important because when *The Cash Report* was entirely redundant, participants could have fully offloaded their decision making by placing 100% weight on *The Cash Report*. If participants were aware they were doing so, reporting accurate SAWs would be easy. In contrast, when *The Cash Report* was only partially redundant, participants had to weigh it against other cues, potentially creating a more difficult metacognitive environment.

We manipulated whether the formula was known or unknown because real school choice websites make their formulas publicly available [58–60], but it is unclear how frequently parents making school choice decisions actually attend to this information. As such, it is important to understand whether or not knowledge of how school ratings are calculated moderates the impact that they have on parents’ metacognitive knowledge of attribute weights. We chose to test this using both attribute sets to ensure that our results were not specific to one attribute set.

### Study 3 analysis & results

#### Estimating revealed attribute weights

Revealed Attribute Weights (RAWs) were estimated from CBC responses using the same techniques as in Studies 1a, 1b, and 2. Mean RAWs, SAWs, and AIRs for each attribute are reported in the S2 Table.

#### Utilization of *The Cash Report*

Participants placed substantial weight on *The Cash Report*, but paired t-tests indicated that RAWs (*M*s = 11.75–17.38) for *The Cash Report* were significantly greater than SAWs (*M*s = 6.31–11.92; *t*s_paired_ = 4.95–11.76, *p*s < .001) in all four experimental conditions. Across the four experimental conditions, zero participants revealed RAWs greater than 50% for *The Cash Report* and only 13/801 participants (1.6%) reported SAWs greater than 50% for *The Cash Report*. This suggests that participants used *The Cash Report* as an additional cue, rather than a heuristic to offload their decision making.

#### Estimating perfect metacognitive knowledge

**Fig 5:**
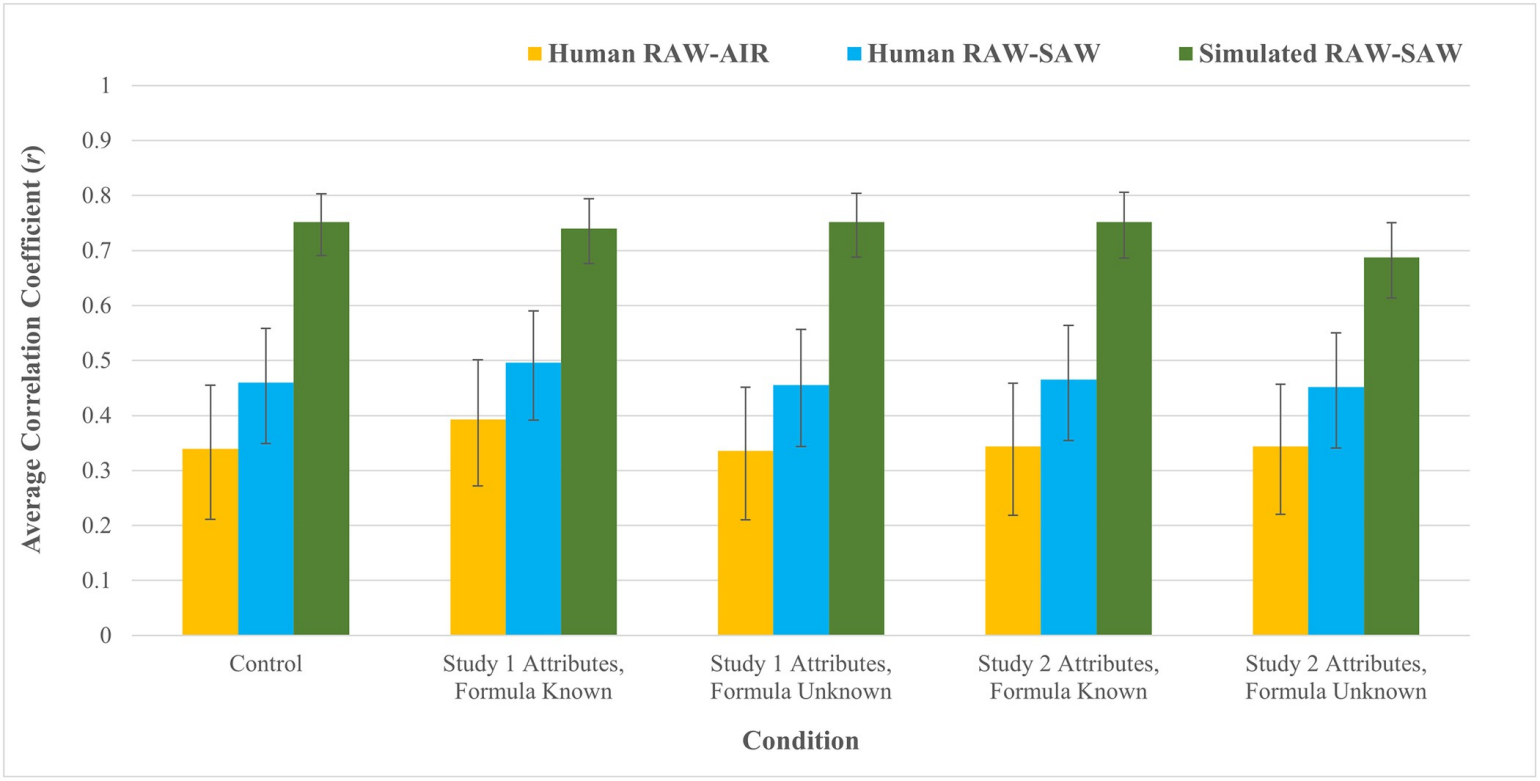
Study 3 average RAW-AIR, RAW-SAW, and simulated RAW-SAW correlations, by condition. Error bars reflect 95% confidence intervals.

As in Studies 1a, 1b, and 2, we estimated perfect metacognitive knowledge via simulated participants. The simulated correlations between assigned SAWs and estimated RAWs for each attribute across the five conditions ranged from *r* = .38 - .90 (see S1 Fig) and the mean correlations (averaged across attributes) for each condition ranged from *r* = .69 - .75 (See Fig 5).

#### RAW-SAW correlations

As in the previous studies, our next step was to evaluate the correlations between participants’ RAWs and SAWs for each attribute. Correlation coefficients for each attribute (reported individually in the S1 Fig) ranged from *r* = .07 - .73 across the five conditions, and the grand mean of the 39 correlation coefficients was *r* = .47. Mean correlations for each condition ranged from *r* = .45 - .50 (see Fig 5). These moderate correlations are on the lower end of the benchmark range for correlations across measurement modalities in the literature (*r* = .40 - .70) [37]. A series of pairwise two-tailed Fisher’s *r* to *z* transformations indicated that the average correlations were not significantly different across conditions (*n*s = 197–203, *z*s = 0.05–0.58, *p*s = .56 - .96), thus suggesting that access to *The Cash Report* did not improve metacognitive knowledge of attribute weights, regardless of whether the formula was (un)known or if *The Cash Report* was redundant to other provided attributes.

We then used two-tailed Fisher’s *r* to *z* transformations to compare the correlations between RAWs and assigned SAWs for the simulated respondents to the correlations between RAWs and SAWs for the human participants for each attribute in each of the five conditions. Correlations for the human participants were significantly lower than correlations for the simulated respondents for 37 of the 39 attributes (*n*s = 197–203, *z*s = -9.97 –-2.04, *p*s = < .001 - .04; see S1 Fig for details). When a Bonferroni correction was applied (α = .05/39 = .0013), the differences remained significant for 33 of the 39 attributes (*n*s = 197–203, *z*s = -9.97 –-2.50, *p*s ≤ .001). Similarly, two-tailed Fisher’s *r* to *z* transformations indicated that the average of the human participants’ correlations was lower than the average of the simulated respondents’ correlations in each of the five conditions, whether a Bonferroni correction (α = .05/5 = .01) was applied or not (*n*s = 197–203, *z*s = -4.81 –-3.58., *p*s < .001; See Fig 5).

#### RAW-SAW different choice predictions

Using the same procedures as Studies 1a, 1b, and 2, we estimated the percentage of decisions (*n*s = 2,758–2,842) for which participants in each condition would have made different choices if they had used their SAWs as attribute weights than they would have if they had used their RAWs as attribute weights. In each condition, we found that weighting by SAWs led to different choices than weighting by RAWs in 18.24% (503/2,758; κ = .69) to 20.63% (569/2,758; κ = .73) of choice tasks. Chi-square tests of equality of proportions indicated that these proportions were not significantly different from one another, *X*^2^ (4, *n*s = 197–203) = 6.29, *p* = .18, suggesting that participants’ metacognitive knowledge was similarly limited regardless of their access to metacognitive aids or how the aids were presented.

#### RAW-AIR correlations

We next evaluated correlations between RAWs and AIRs. Correlation coefficients for each attribute (see S1 Fig) ranged from *r* = .11 - .55 across the five conditions, and the grand mean of the 39 correlation coefficients was *r* = .35. Mean correlations for each condition ranged from *r* = .34 - .39 (see Fig 5). These low-to-moderate correlations fall slightly below the benchmark range (*r* = .40 - .70) [37] for correlations between attribute weights measured via different elicitation methods. Furthermore, a series of pairwise two-tailed Fisher’s *r* to *z* transformations indicated that the average correlations were not significantly different across conditions (*n*s = 197–203, *z*s = 0.00–0.64, *p*s = .52 –.999), once again suggesting that access to *The Cash Report* did not affect participants’ metacognitive knowledge of the attributes they used when making their school choice decisions. A series of Fisher’s *r* to *z* transformations indicated that mean RAW-SAW and RAW-AIR correlations were not significantly different from one another within any condition (*n*s = 197–203, *z*s = -1.44 –-1.28, *p*s = .15 - .20).

#### Demographic differences in metacognitive knowledge

We then regressed participants’ Average RAW-SAW Differences (descriptive statistics reported in the S2 Table) on the same six demographic variables as in the previous studies. When aggregating subjects from all conditions into a single regression, only the coefficient for college education was significant (*B* = -0.74, *SE* = 0.23 *p* = .001). The full regression is reported in Table 4. Given the inconsistency in significant effects across studies 1a, 1b, 2, and 3, we observe that demographic characteristics are not reliable predictors of metacognitive knowledge of attribute weights. Separate regressions for each condition are reported in the S3 Table. Comparisons of mean Average RAW-SAW Differences across conditions are reported in the S1 Text.

**Table 4:** Study 3 demographic factors predicting average RAW-SAW differences.

Dependent Variable: Average RAW-SAW Difference		Study 3 *R*^2^ = .03		
	*B*	*95% CI*	*SE*	*p*
Intercept	9.97***	8.89–11.05	0.55	< .001
Female	0.16	-0.26–0.59	0.22	.45
Age	-0.02	-0.03–0.00	0.01	.08
Suburban	-0.30	-0.78–0.18	0.25	.22
Rural	0.05	-0.60–0.70	0.33	.88
Non-Parent	-0.44	-0.90–0.03	0.24	.07
Bachelor’s Degree	-0.74**	-1.19 –-0.29	0.23	.001
Income	-0.08	-0.20–0.05	0.06	.23

Note.

*p < .05,

**p < .01,

***p < .001;

Male, Urban, Parent, and No Bachelor’s Degree were used as comparison groups; Similar regressions that used Rural as the comparison group indicated no significant effects of urban status.

### Study 3 discussion

Study 3 replicated the findings from Studies 1a, 1b, and 2 that individuals’ metacognitive knowledge of the weight they place on various attributes is significantly limited in school choice decisions. This finding was once again robust to two different stated preference response formats (AIRs and SAWs). Study 3 also provided additional evidence that demographic characteristics do not consistently predict participants’ metacognitive knowledge of attribute weights. Most importantly, however, Study 3 demonstrated that providing decision makers with access to an aggregate school rating does not improve their metacognitive knowledge. Instead, decision makers simply treated the aggregate rating as one more cue to incorporate into their weighting scheme. This extension of our findings suggests that the tools currently available to parents may be insufficient to help them overcome their metacognitive limitations.

## General discussion

Across four experiments, we found that participants who were tasked with making school choice decisions displayed a lack of metacognitive knowledge of the attribute weights they had used. The degree of miscalibration was similar to other domains studied in the literature [37], suggesting that school choice, despite having characteristics that might lead one to expect better metacognitive calibration, is not a domain in which decision makers have a uniquely high degree of metacognitive knowledge. This finding held true regardless of whether the participants were a general online sample (Study 1a) or a sample of parents of high school-aged children (Study 1b) and whether the attributes were primarily academic in nature (Studies 1a and 1b) or a diverse combination of academic and non-academic attributes (Study 2). The result also persisted when participants were given access to an aggregate school rating (Study 3). These results were robust to multiple preference elicitation methods and participant-level metacognitive calibration was not consistently predicted by demographic characteristics. This pattern of results suggests a fairly robust phenomenon that decision makers lack metacognitive knowledge of their preferences when making school choice decisions.

### Implications

These results–which align with decades of psychological research [31, 37]–undermine the assumption held by most school choice policies that parents can accurately self-assess the importance of various school attributes. Though our results were obtained from hypothetical school choice decisions, we contend that they are likely to generalize to real school choice decisions because real school choice decisions are even more complex than the choices participants made in our studies. In more naturalistic school choice environments, parents are expected to make choices between dozens of schools, each of which has tens, if not hundreds, of attributes that must be weighted. Our results show that, even in a simplified school choice decision environment (with only seven or eight attributes and three schools), participants showed poor metacognitive calibration. As such, our results provide suggestive evidence that parents lack the necessary metacognitive capacities to truly take advantage of school choice policies that require them to make highly complex, metacognitively demanding decisions.

Policymakers should consider these limitations and design school choice policies in ways that limit the need for parents to make decisions that rely on extensive metacognitive knowledge of their own attribute weights. Because we appreciate the complexity of the policymaking process in a domain as sensitive as education, we will not offer a prescriptive, one-size-fits all solution. Instead, we encourage policymakers to develop solutions that are tailored to the goals, interests, and beliefs of the parents and other stakeholders in their community [15, 16, 42–45]. However, we suggest that any policy seeking to reduce metacognitive burden should, at its core, focus on solutions that make school choice decisions easier by reducing the amount of information that parents must evaluate [18]. Regardless of the specific policy solution that works in any given community, the key takeaway from our results is that policymakers should consider metacognitive limitations as a potential barrier to the success of school choice policies going forward.

These results also have implications for interpreting polling data about education. Americans have consistently reported low levels of satisfaction with the K-12 education system [1]. One reason for this dissatisfaction may be that parents do not have metacognitive access to their preferences. If parents express certain goals and preferences that differ from what truly leads them to be satisfied with schools, then responsive policy makers and educators may intervene on the wrong attributes. Given our findings, educators may not be able to take input and feedback from parents at face value, which could impact their ability to optimally allocate scarce education resources. Though we encourage educators to continue seeking feedback from stakeholders, our results highlight the importance of seeking this feedback in formats that are intuitive and less dependent on accurate metacognitive knowledge of attribute weights.

Our results build upon decades of psychological research showing that participants have limited metacognitive knowledge of the factors that influence their judgments and decisions [31, 37] by replicating the finding in the domain of school choice. This is particularly important because school choice is a domain in which it would be reasonable–and common in policy circles [2–8]–to believe that decision makers might have unique insight into their preferences, due to the domain’s cultural and personal importance and salience [43]. As such, our results suggest that metacognitive limitations in decision making may be more pervasive than previously believed.

### Limitations

Our studies had two key limitations. First, the choices made by participants were hypothetical, and thus may have lacked some aspects of real-world decisions that could promote metacognitive knowledge of attribute weights. As such, we cannot rule out the possibility that parents making real school choice decisions would display greater metacognitive knowledge than participants making hypothetical school choice decisions. Though we demonstrated in Study 1a and Study 1b that parents and non-parents show similar levels of metacognitive knowledge when making hypothetical school choice decisions, it is possible that parents who make numerous real school choice decisions may develop better metacognitive knowledge through experience. Furthermore, parents making real school choice decisions are also likely to be more motivated to make accurate choices than participants making hypothetical choices. However, real-world decisions are also plagued by complexities, such as incomplete information, large choice sets, and extensive attribute lists, that could impair metacognitive knowledge to an even greater extent than was demonstrated in our results. Additionally, the cognitive psychology literature suggests that incentives alone (i.e., without other interventions, such as feedback) are often insufficient to improve metacognitive accuracy [66, 67]. As such, we contend that our findings provide valuable insights into metacognitive knowledge in the school choice context, but encourage future research that seeks to generalize our findings in more naturalistic contexts.

Second, we were only able to test a small number of the many attributes that parents may evaluate when comparing schools; while we examined both academic and non-academic factors, we were unable to explore attributes that were less quantifiable, such as a parent’s emotional reaction to a school, or the school’s culture. As such, we cannot confirm that parents would have similarly limited metacognitive knowledge for all possible school choice attributes. Furthermore, it is plausible that parents may have a strong metacognitive sense for when a school *feels* right but may not be able to justify the feeling in terms of attributes. It is also possible that parents may evaluate interactions between attributes (e.g., high graduation rates are only meaningful if test scores are also high), which we could not capture in this study. Despite these limitations, we argue that our results maintain relevance, as the individual quantifiable attributes we chose reflect the attributes that are most often considered by parents [40] and by experts who design public policies (e.g., No Child Left Behind) and educational resources (e.g., U.S. News & World Report) [58–60]. However, we encourage future research exploring metacognitive knowledge of attribute weights in the school choice context using different attribute sets.

### Future research

In addition to addressing the limitations of this study, future research should seek to expand upon our findings by exploring how school choice policies and processes could be altered to make it easier for parents to develop and implement accurate metacognitive knowledge. This could be achieved by changing the decision environment itself–such as by reducing the number of attributes or alternatives–or through targeted interventions. One class of interventions that the literature suggests may be particularly promising includes interventions that make it easier for parents to digest the information they are given, often by reducing or summarizing the information [18]. It is important for this research to be conducted among diverse samples so that we can develop a thorough and nuanced understanding of how effectively different solutions may function in communities with different goals, needs, and beliefs [15, 16, 42–45].

Another interesting avenue for future research would be to explore individual difference variables that may influence metacognitive knowledge of attribute weights in the school choice context. Though we explored basic demographic characteristics here, it is likely that other psychological characteristics–such as intelligence [68] or numeracy [69]–may influence metacognitive knowledge of attribute weights more directly. Understanding the psychological characteristics that predict metacognitive knowledge in the school choice context is interesting not only theoretically, but practically as well, as this knowledge could be used to help policymakers target interventions at the individuals that need them most. In all, we believe that metacognitive knowledge of attribute weights in the school choice context is a fruitful domain for future research.

## Conclusions

Over four studies, we found that participants lacked metacognitive knowledge of the attributes that influenced their school choice decisions. By showing that decision makers lack the necessary metacognitive capacity to make optimal school choice decisions, our research provides a novel explanation for the relative inefficacy of school choice policies as currently instantiated. Because parents lack metacognitive knowledge of the weight they place on various school choice attributes, they may be more likely to pick schools that do not match their true preferences. This is particularly likely in the complex, multi-attribute choice context in which school choice decisions are often presented, where parents must compare dozens of schools in terms of tens, if not hundreds, of attributes. As such, policymakers should consider alternative approaches to school choice that account for parents’ metacognitive limitations. Our findings also contribute to the metacognition literature by showing that classic psychological findings demonstrating decision makers’ lack of metacognitive knowledge of the factors that influence their decisions extends to the highly salient and culturally important domain of education–a result that may be surprising to policymakers and educational advocates who have argued that parents are the best judges of their families’ educational needs. Future research should seek to test different approaches to school choice to determine how school choice policies can be designed to help parents make better decisions despite their metacognitive limitations and should seek to evaluate individual difference variables that may be associated with better or worse metacognitive knowledge so that school choice interventions can be optimally targeted. In all, our findings suggest that school choice initiatives may need to worry less about parents’ rights and more about parents’ (metacognitive) wrongs.

## Supporting information

S1 Table Demographic characteristics for each study and condition. (DOCX)

S2 Table RAW, SAW, AIR, and RAW-SAW Difference descriptive statistics for each study and condition. (DOCX)

S3 Table Demographic factors predicting average RAW-SAW Differences for each Study 3 condition. (DOCX)

S1 Fig Attribute-level RAW-SAW, RAW-AIR, and simulated RAW-SAW correlations for each Study 3 condition. (DOCX)

S1 Appendix Attribute descriptions and levels. (DOCX)

S2 Appendix Pilot studies. (DOCX)

S1 Text Comparison of mean average RAW-SAW differences across Study 3 conditions. (DOCX)

S1 Checklist Human participants research checklist. (DOCX)
