# Supplementary material for: Parental rights or parental wrongs: Parents’ metacognitive knowledge of the factors that influence their school choice decisions
Source: PLoS One. 2024 Apr 18;19(4):e0301768. doi: 10.1371/journal.pone.0301768 (PMC11025896; doi:10.1371/journal.pone.0301768)
Supplement: S1 Table — (DOCX) [file pone.0301768.s001.docx]

| **Variable** | **Study 1a**  **(*n* = 191)** | **Study 1b**  **(*n* = 202)** | **Study 2**  **(*n* = 214)** |
| --- | --- | --- | --- |
| Females | 86 (45.0%) | 121 (59.9%) | 95 (44.4%) |
| Average Age (*SD*) | 38.6 (10.7) | 44.9 (7.8) | 39.1 (11.5) |
| Race |  |  |  |
| White/Caucasian | 165 (86.4%) | 167 (82.7%) | 174 (81.3%) |
| Black/African American | 12 (6.3%) | 19 (9.4%) | 16 (7.5%) |
| Asian/Asian American | 6 (3.1%) | 7 (3.5%) | 19 (8.9%) |
| Native American/Native Alaskan | 1 (0.5%) | 0 (0.0%) | 0 (0.0%) |
| Multi-Racial/Other Race | 5 (2.3%) | 9 (4.5%) | 3 (1.4%) |
| Hispanic/Latinx | 9 (4.7%) | 12 (5.9%) | 14 (6.5%) |
| Urban Status |  |  |  |
| Urban | 50 (26.2%) | 58 (28.7%) | 55 (25.7%) |
| Suburban | 99 (51.8%) | 106 (52.5%) | 122 (57.0%) |
| Rural | 42 (22.0%) | 38 (18.8%) | 36 (16.8%) |
| Parents/Have Children | 78 (40.8%) | 202 (100.0%) | 90 (42.1%) |
| Avg. Number of Children (*SD*)^a^ | 2.2 (1.2) | 2.1 (1.1) | 2.0 (0.8) |
| Avg. Age of Youngest Child (*SD*)^a^ | 12.4 (10.3) | 11.5 (5.6) | 13.0 (10.6) |
| Avg. Age of Oldest Child (*SD*)^a^ | 16.9 (11.3) | 16.2 (1.5) | 16.4 (10.6) |
| Bachelor’s Degree | 110 (57.6%) | 129 (63.9%) | 122 (57.0%) |
| Annual Household Income |  |  |  |
| $0 - $24,999 | 34 (17.8%) | 19 (9.4%) | 27 (12.6%) |
| $25,000 - $49,999 | 48 (25.1%) | 28 (13.9%) | 52 (24.3%) |
| $50,000 - $74,999 | 52 (27.2%) | 45 (22.2%) | 47 (22.0%) |
| $75,000 - $99,999 | 33 (17.3%) | 35 (17.3%) | 32 (15.0%) |
| $100,000 - $124,999 | 6 (3.1%) | 17 (8.4%) | 12 (5.6%) |
| $125,000 - $149,999 | 8 (4.2%) | 27 (13.4%) | 14 (6.5%) |
| $150,000 - $174,999 | 6 (3.1%) | 9 (4.5%) | 7 (3.3%) |
| $175,000 - $200,000 | 0 (0.0%) | 3 (1.5%) | 7 (3.3%) |
| $200,000 or Greater | 4 (2.1%) | 16 (7.9%) | 10 (4.7%) |

**Study 1a, 1b, and 2 Demographic Characteristics**

^a^Calculated based on participants with children only

| **Variable** | **Control**  **(*n* = 197)** | **S1K**  **(*n* = 201)** | **S1UK**  **(*n* = 200)** |
| --- | --- | --- | --- |
| Females | 107 (54.3%) | 101 (50.2%) | 98 (49.0%) |
| Average Age (*SD*) | 38.0 (11.1) | 40.5 (13.1) | 39.1 (12.1) |
| Race |  |  |  |
| White/Caucasian | 146 (74.1%) | 156 (77.6%) | 154 (77.0%) |
| Black/African American | 21 (10.7%) | 16 (8.0%) | 17 (8.5%) |
| Asian/Asian American | 16 (8.1%) | 15 (7.5%) | 17 (8.5%) |
| Native American/Native Alaskan | 2 (1.0%) | 3 (1.5%) | 0 (0.0%) |
| Multi-Racial/Other Race | 11 (6.1%) | 10 (5.0%) | 10 (5.0%) |
| Hispanic/Latinx | 9 (5.6%) | 15 (7.5%) | 14 (7.0%) |
| Urban Status |  |  |  |
| Urban | 52 (26.4%) | 57 (28.4%) | 63 (31.5%) |
| Suburban | 116 (58.9%) | 107 (53.2%) | 104 (52.0%) |
| Rural | 29 (14.7%) | 36 (17.9%) | 32 (16.0%) |
| Parents/Have Children | 96 (48.7%) | 91 (45.3%) | 87 (43.5%) |
| Avg. Number of Children (*SD*)^a^ | 2.0 (1.1) | 2.0 (1.1) | 2.2 (1.2) |
| Avg. Age of Youngest Child (*SD*)^a^ | 12.2 (9.7) | 14.0 (10.4) | 13.5 (11.4) |
| Avg. Age of Oldest Child (*SD*)^a^ | 16.1 (10.5) | 17.9 (10.5) | 18.0 (12.7) |
| Bachelor’s Degree | 111 (56.3%) | 127 (63.2%) | 113 (56.5%) |
| Annual Household Income |  |  |  |
| $0 - $24,999 | 20 (10.1%) | 30 (14.9%) | 21 (10.5%) |
| $25,000 - $49,999 | 53 (26.9%) | 48 (23.9%) | 58 (29.0%) |
| $50,000 - $74,999 | 45 (22.8%) | 42 (20.9%) | 45 (22.5%) |
| $75,000 - $99,999 | 50 (25.4%) | 40 (19.9%) | 36 (18%) |
| $100,000 - $124,999 | 11 (5.6%) | 18 (9.0%) | 17 (8.5%) |
| $125,000 - $149,999 | 6 (3.0%) | 10 (5.0%) | 7 (3.5%) |
| $150,000 - $174,999 | 5 (2.5%) | 0 (0.0%) | 4 (2.0%) |
| $175,000 - $200,000 | 2 (1.0%) | 4 (2.0%) | 4 (2.0%) |
| $200,000 or Greater | 3 (1.5%) | 4 (2.0%) | 6 (3.0%) |

**Study 3 Demographic Characteristics, By Condition**

^a^Calculated based on participants with children only

| **Variable** | **S2K**  **(*n* = 197)** | **S2UK**  **(*n* = 203)** |
| --- | --- | --- |
| Females | 97 (49.2%) | 101 (49.8%) |
| Average Age (*SD*) | 41.1 (12.9) | 38.5 (12.0) |
| Race |  |  |
| White/Caucasian | 156 (79.2%) | 157 (77.3%) |
| Black/African American | 19 (9.6%) | 18 (8.9%) |
| Asian/Asian American | 15 (7.6%) | 12 (5.9%) |
| Native American/Native Alaskan | 1 (0.5%) | 1 (0.5%) |
| Multi-Racial/Other Race | 5 (2.5%) | 14 (6.9%) |
| Hispanic/Latinx | 15 (7.6%) | 18 (8.9%) |
| Urban Status |  |  |
| Urban | 55 (27.9%) | 52 (25.6%) |
| Suburban | 104 (52.8%) | 115 (56.7%) |
| Rural | 37 (18.8%) | 35 (17.2%) |
| Parents/Have Children | 100 (50.8%) | 102 (50.2%) |
| Avg. Number of Children (*SD*)^a^ | 2.1 (1.2) | 2.1 (1.0) |
| Avg. Age of Youngest Child (*SD*)^a^ | 14.3 (12.3) | 12.9 (10.0) |
| Avg. Age of Oldest Child (*SD*)^a^ | 18.2 (13.6) | 17.3 (11.1) |
| Bachelor’s Degree | 120 (60.9%) | 118 (58.1%) |
| Annual Household Income |  |  |
| $0 - $24,999 | 27 (13.7%) | 19 (9.4%) |
| $25,000 - $49,999 | 57 (28.9%) | 59 (29.1%) |
| $50,000 - $74,999 | 45 (22.8%) | 44 (21.7%) |
| $75,000 - $99,999 | 26 (13.2%) | 39 (19.2%) |
| $100,000 - $124,999 | 13 (6.6%) | 13 (6.4%) |
| $125,000 - $149,999 | 12 (6.1%) | 15 (7.4%) |
| $150,000 - $174,999 | 8 (4.1%) | 6 (3.0%) |
| $175,000 - $200,000 | 1 (0.5%) | 0 (0.0%) |
| $200,000 or Greater | 6 (3.0%) | 6 (3.0%) |

**Study 3 Demographic Characteristics, By Condition (cont’d)**

^a^Calculated based on participants with children only
