# Supplementary material for: Parental rights or parental wrongs: Parents’ metacognitive knowledge of the factors that influence their school choice decisions
Source: PLoS One. 2024 Apr 18;19(4):e0301768. doi: 10.1371/journal.pone.0301768 (PMC11025896; doi:10.1371/journal.pone.0301768)
Supplement: S2 Table — (DOCX) [file pone.0301768.s002.docx]

**Studies 1a, 1b, and 2 Mean RAWs (with Standard Deviations) for Each Attribute**

| Attribute | Study 1a | Study 1b | Study 2 |
| --- | --- | --- | --- |
| AP Enrollment | 8.05 (3.36) | 7.73 (2.72) | - |
| Average ACT Score | 16.27 (9.77) | 16.12 (9.83) | - |
| Average Parent Rating | 22.48 (11.53) | 25.64 (12.59) | 19.71 (11.91) |
| Crime Rate | - | - | 17.69 (11.37) |
| Disadvantaged Student Gap | 7.61 (2.91) | 7.43 (3.40) | - |
| Emotional Support Score | - | - | 10.77 (3.87) |
| Graduation Rate | 14.96 (8.28) | 14.14 (8.71) | 15.23 (8.81) |
| Percent Minority Students | 12.58 (7.15) | 13.61 (7.51) | - |
| Per-Student Spending | - | - | 7.04 (3.38) |
| State Test Pass Rate | 18.06 (10.94) | 15.34 (7.77) | 21.80 (12.96) |
| Teacher Exam Score | - | - | 7.76 (3.86) |

| Attribute | Study 3 CTR | Study 3  S1K | Study 3  S1UK | Study 3  S2K | Study 3  S2UK |
| --- | --- | --- | --- | --- | --- |
| AP Enrollment | 6.38 (2.63) | 6.89 (2.17) | 6.26 (2.21) | - | - |
| Average ACT Score | 19.89 (11.02) | 16.25 (11.53) | 14.70 (8.59) | - | - |
| Average Parent Rating | 22.88 (11.75) | 20.03 (10.43) | 22.63 (11.77) | 16.34 (6.54) | 17.98 (8.74) |
| Crime Rate | - | - | - | 14.31 (7.13) | 16.72 (10.39) |
| Disadvantaged Student Gap | 7.36 (3.56) | 6.40 (2.73) | 6.20 (2.88) | - | - |
| Emotional Support Score | - | - | - | 10.29 (5.45) | 9.92 (3.89) |
| Graduation Rate | 15.08 (8.47) | 11.57 (6.80) | 10.98 (4.78) | 10.21 (5.00) | 9.14 (3.84) |
| Percent Minority Students | 11.33 (6.27) | 9.27 (4.86) | 11.01 (6.48) | - | - |
| Per-Student Spending | - | - | - | 7.34 (3.14) | 5.98 (2.44) |
| State Test Pass Rate | 17.07 (10.05) | 12.20 (5.87) | 12.57 (6.01) | 20.60 (12.83) | 20.74 (11.62) |
| Teacher Exam Score | - | - | - | 7.52 (3.22) | 7.76 (3.54) |
| Cash Report | - | 17.38 (8.21) | 15.66 (7.03) | 13.39 (5.73) | 11.75 (5.29) |

**Study 3 Mean RAWs (with Standard Deviations) for Each Attribute, by Condition**

*Note. CTR = Control; S1 = Study 1 Attributes; S2 = Study 2 Attributes; K = Known Formula; UK = Unknown Formula*

**Studies 1a, 1b, and 2 Mean SAWs (with Standard Deviations) for Each Attribute**

| Attribute | Study 1a | Study 1b | Study 2 |
| --- | --- | --- | --- |
| AP Enrollment | 7.73 (7.81) | 8.25 (9.95) | - |
| Average ACT Score | 17.91 (15.84) | 16.77 (15.71) | - |
| Average Parent Rating | 17.00 (16.77) | 19.48 (19.19) | 12.42 (14.13) |
| Crime Rate | - | - | 16.83 (15.90) |
| Disadvantaged Student Gap | 5.87 (7.17) | 5.87 (7.74) | - |
| Emotional Support Score | - | - | 7.87 (7.27) |
| Graduation Rate | 26.90 (17.82) | 24.02 (16.95) | 27.48 (16.61) |
| Percent Minority Students | 9.01 (13.93) | 11.40 (15.00) | - |
| Per-Student Spending | - | - | 8.08 (7.24) |
| State Test Pass Rate | 15.59 (12.29) | 14.21 (10.60) | 18.14 (13.18) |
| Teacher Exam Score | - | - | 9.19 (8.29) |

**Study 3 Mean SAWs (with Standard Deviations) for Each Attribute, by Condition**

| Attribute | Study 3 CTR | Study 3  S1K | Study 3  S1UK | Study 3  S2K | Study 3  S2UK |
| --- | --- | --- | --- | --- | --- |
| AP Enrollment | 7.75 (5.95) | 6.85 (6.28) | 6.69 (6.02) | - | - |
| Average ACT Score | 17.85 (17.38) | 17.11 (16.94) | 15.57 (13.52) | - | - |
| Average Parent Rating | 16.06 (17.91) | 15.46 (16.11) | 17.49 (19.10) | 11.71 (11.30) | 11.92 (12.50) |
| Crime Rate | - | - | - | 15.48 (14.80) | 16.67 (13.57) |
| Disadvantaged Student Gap | 5.46 (6.43) | 4.67 (5.32) | 4.97 (5.50) | - | - |
| Emotional Support Score | - | - | - | 9.73 (10.13) | 10.11 (10.21) |
| Graduation Rate | 28.95 (18.83) | 23.14 (17.04) | 23.45 (17.31) | 22.71 (15.40) | 22.23 (14.84) |
| Percent Minority Students | 8.79 (10.59) | 8.00 (11.48) | 9.65 (14.51) | - | - |
| Per-Student Spending | - | - | - | 6.65 (6.26) | 6.54 (6.45) |
| State Test Pass Rate | 15.14 (12.72) | 12.86 (10.92) | 12.56 (11.01) | 16.87 (13.68) | 18.72 (14.58) |
| Teacher Exam Score | - | - | - | 7.67 (8.07) | 7.51 (6.80) |
| Cash Report | - | 11.92 (14.70) | 9.63 (13.63) | 9.20 (13.45) | 6.31 (6.13) |

*Note. CTR = Control; S1 = Study 1 Attributes; S2 = Study 2 Attributes; K = Known Formula; UK = Unknown Formula*

**Studies 1a, 1b, and 2 Mean AIRs (with Standard Deviations) for Each Attribute**

| Attribute | Study 1a | Study 1b | Study 2 |
| --- | --- | --- | --- |
| AP Enrollment | 4.89 (2.21) | 5.09 (2.10) | - |
| Average ACT Score | 6.35 (1.99) | 6.39 (1.93) | - |
| Average Parent Rating | 6.09 (2.35) | 6.51 (2.26) | 5.50 (2.38) |
| Crime Rate | - | - | 6.52 (2.04) |
| Disadvantaged Student Gap | 4.30 (2.09) | 4.53 (2.21) | - |
| Emotional Support Score | - | - | 4.99 (2.20) |
| Graduation Rate | 7.30 (1.84) | 7.07 (1.82) | 7.81 (1.32) |
| Percent Minority Students | 4.16 (2.57) | 4.64 (2.70) | - |
| Per-Student Spending | - | - | 5.08 (2.06) |
| State Test Pass Rate | 6.29 (2.05) | 6.32 (1.92) | 6.71 (2.00) |
| Teacher Exam Score | - | - | 5.68 (1.98) |

**Study 3 Mean AIRs (with Standard Deviations) for Each Attribute, by Condition**

| Attribute | Study 3 CTR | Study 3  S1K | Study 3  S1UK | Study 3  S2K | Study 3  S2UK |
| --- | --- | --- | --- | --- | --- |
| AP Enrollment | 5.08 (2.08) | 4.95 (2.13) | 4.81 (2.09) | - | - |
| Average ACT Score | 6.44 (1.90) | 6.38 (2.02) | 6.23 (1.95) | - | - |
| Average Parent Rating | 5.81 (2.41) | 6.02 (2.28) | 5.94 (2.35) | 5.61 (2.39) | 5.74 (2.27) |
| Crime Rate | - | - | - | 6.23 (2.08) | 6.65 (2.14) |
| Disadvantaged Student Gap | 4.30 (2.14) | 4.23 (2.11) | 4.12 (2.19) | - | - |
| Emotional Support Score | - | - | - | 5.35 (2.28) | 5.48 (2.37) |
| Graduation Rate | 7.61 (1.46) | 7.13 (1.85) | 7.11 (1.75) | 7.24 (1.57) | 7.34 (1.68) |
| Percent Minority Students | 4.37 (2.54) | 4.17 (2.47) | 4.52 (2.59) | - | - |
| Per-Student Spending | - | - | - | 4.71 (2.00) | 4.79 (2.21) |
| State Test Pass Rate | 6.32 (2.06) | 6.22 (1.83) | 6.26 (1.78) | 6.49 (2.06) | 6.69 (2.17) |
| Teacher Exam Score | - | - | - | 5.30 (2.05) | 5.29 (2.23) |
| Cash Report | - | 4.92 (2.22) | 4.34 (2.17) | 4.37 (2.11) | 4.22 (2.09) |

*Note. CTR = Control; S1 = Study 1 Attributes; S2 = Study 2 Attributes; K = Known Formula; UK = Unknown Formula*

**Studies 1a, 1b, and 2 Mean RAW-SAW Differences (with Standard Deviations) for Each Attribute**

| Attribute | Study 1a | Study 1b | Study 2 |
| --- | --- | --- | --- |
| AP Enrollment | 6.39 (4.77) | 6.22 (7.10) | - |
| Average ACT Score | 8.89 (7.90) | 8.24 (7.25) | - |
| Average Parent Rating | 10.35 (8.67) | 11.85 (9.25) | 10.44 (8.11) |
| Crime Rate | - | - | 7.49 (6.84) |
| Disadvantaged Student Gap | 5.38 (4.82) | 5.16 (4.94) | - |
| Emotional Support Score | - | - | 6.43 (4.31) |
| Graduation Rate | 14.77 (12.25) | 13.52 (11.79) | 14.09 (11.92) |
| Percent Minority Students | 7.99 (7.68) | 8.99 (7.78) | - |
| Per-Student Spending | - | - | 5.75 (4.92) |
| State Test Pass Rate | 7.72 (6.56) | 7.21 (6.18) | 8.67 (7.18) |
| Teacher Exam Score | - | - | 5.71 (6.03) |
| Cash Report | - | - | - |
| Average | **8.79 (3.32)** | **8.74 (3.34)** | **8.79 (3.39)** |

**Study 3 Mean RAW-SAW Differences (with Standard Deviations) for Each Attribute, by Condition**

| Attribute | Study 3 CTR | Study 3  S1K | Study 3  S1UK | Study 3  S2K | Study 3  S2UK |
| --- | --- | --- | --- | --- | --- |
| AP Enrollment | 5.23 (3.81) | 5.01 (3.92) | 4.74 (3.48) | - | - |
| Average ACT Score | 9.68 (8.54) | 8.06 (8.27) | 7.83 (6.97) | - | - |
| Average Parent Rating | 12.06 (9.51) | 10.40 (7.84) | 11.95 (9.67) | 8.30 (5.88) | 9.15 (6.94) |
| Crime Rate | - | - | - | 7.69 (9.07) | 7.62 (7.31) |
| Disadvantaged Student Gap | 5.34 (4.13) | 4.64 (3.26) | 4.76 (3.29) | - | - |
| Emotional Support Score | - | - | - | 5.86 (5.06) | 6.57 (6.07) |
| Graduation Rate | 16.36 (14.59) | 13.16 (12.72) | 14.71 (14.21) | 14.51 (12.40) | 14.20 (12.28) |
| Percent Minority Students | 6.85 (5.55) | 6.75 (6.68) | 7.87 (8.60) | - | - |
| Per-Student Spending | - | - | - | 5.34 (4.05) | 4.75 (4.70) |
| State Test Pass Rate | 8.93 (7.10) | 6.96 (6.74) | 6.82 (6.58) | 8.55 (7.24) | 8.56 (6.98) |
| Teacher Exam Score | - | - | - | 5.46 (5.74) | 5.32 (4.20) |
| Cash Report | - | 9.96 (8.53) | 10.15 (9.40) | 8.52 (9.28) | 7.08 (4.81) |
| Average | **9.21 (3.55)** | **8.12** **(3.13)** | **8.60 (3.56)** | **8.03 (3.27)** | **7.91 (2.91)** |

*Note. CTR = Control; S1 = Study 1 Attributes; S2 = Study 2 Attributes; K = Known Formula; UK = Unknown Formula*
