# Supplementary material for: Parental rights or parental wrongs: Parents’ metacognitive knowledge of the factors that influence their school choice decisions
Source: PLoS One. 2024 Apr 18;19(4):e0301768. doi: 10.1371/journal.pone.0301768 (PMC11025896; doi:10.1371/journal.pone.0301768)
Supplement: S3 Table — (DOCX) [file pone.0301768.s003.docx]

**Study 3 Demographics Predicting Average RAW-SAW Differences, by Condition**

| **Dependent Variable:**  Average RAW-SAW Difference | **CTR**  *R*^2^ = .06 | | | |
| --- | --- | --- | --- | --- |
|  | *B* | *95% CI* | *SE* | *p* |
| Intercept | 11.79*** | 9.05 – 14.54 | 1.39 | <.001 |
| Female | 0.31 | -0.70 – 1.32 | 0.51 | .55 |
| Age | -0.04 | -0.09 – 0.01 | 0.02 | .10 |
| Suburban | -0.38 | -1.55 – 0.78 | 0.59 | .52 |
| Rural | 0.14 | -1.51 – 1.79 | 0.84 | .87 |
| Non-Parent | -1.12 | -2.24 – 0.01 | 0.57 | .05 |
| Bachelor’s Degree | -1.07* | -2.11 – -0.03 | 0.53 | .04 |
| Income | 0.01 | -0.31 – 0.33 | 0.16 | .95 |

*Note*. **p*<.05, ***p*<.01, ****p* < .001; Male, Urban, Parent, and No Bachelor’s Degree were used as comparison groups; Similar regressions that used Rural as the comparison group indicated no significant effects of urban status.

**Study 3 Demographics Predicting Average RAW-SAW Differences, by Condition (cont’d)**

| **Dependent Variable:**  Average RAW-SAW Difference |  | **S1K**  *R*^2^ = .03 | | | **S1UK**  *R*^2^ = .05 | | | | |
| --- | --- | --- | --- | --- | --- | --- | --- | --- | --- |
|  | *B* | *95% CI* | *SE* | *p* | | *B* | *95% CI* | *SE* | *p* |
| Intercept | 8.48*** | 6.11 – 10.84 | 1.20 | <.001 | | 12.16*** | 9.49 – 14.83 | 1.35 | <.001 |
| Female | 0.14 | -0.80 – 1.09 | 0.48 | .77 | | -0.34 | -1.39 – 0.70 | 0.53 | .52 |
| Age | 0.01 | -0.03 – 0.05 | 0.02 | .53 | | -0.05* | -0.09 – 0.00 | 0.02 | .03 |
| Suburban | -0.34 | -1.41 – 0.73 | 0.54 | .53 | | -0.21 | -1.36 – 0.95 | 0.59 | .73 |
| Rural | 0.11 | -1.32 – 1.54 | 0.73 | .88 | | -0.08 | -1.65 – 1.49 | 0.80 | .92 |
| Non-Parent | -0.03 | -1.08 – 1.02 | 0.53 | .96 | | -0.76 | -1.88 – 0.35 | 0.57 | .18 |
| Bachelor’s Degree | -0.67 | -1.73 – 0.38 | 0.53 | .21 | | -0.79 | -1.91 – 0.33 | 0.57 | .17 |
| Income | -0.10 | -0.39 – 0.20 | 0.15 | .51 | | -0.16 | -0.46 – 0.14 | 0.15 | .30 |

*Note*. **p*<.05, ***p*<.01, ****p* < .001; Male, Urban, Parent, and No Bachelor’s Degree were used as comparison groups; Similar regressions that used Rural as the comparison group indicated no significant effects of urban status.

**Study 3 Demographics Predicting Average RAW-SAW Differences, by Condition (cont’d)**

| **Dependent Variable:**  Average RAW-SAW Difference | **S2K**  *R*^2^ = .04 | | | | **S2UK**  *R*^2^ = .07 | | | |  |
| --- | --- | --- | --- | --- | --- | --- | --- | --- | --- |
|  | *B* | *95% CI* | *SE* | *p* | *B* | *95% CI* | *SE* | *p* | |
| Intercept | 9.63*** | 7.30 – 11.96 | 1.18 | <.001 | 7.55*** | 5.43 – 9.67 | 1.08 | <.001 | |
| Female | -0.19 | -1.14 – 0.75 | 0.48 | .69 | 0.66 | -0.16 – 1.48 | 0.42 | .12 | |
| Age | -0.02 | -0.06 – 0.02 | 0.02 | .28 | 0.03 | -0.01 – 0.06 | 0.02 | .17 | |
| Suburban | 0.37 | -0.72 – 1.45 | 0.55 | .51 | -1.40** | -2.37 – -0.43 | 0.49 | .005 | |
| Rural | 0.55 | -0.87 – 1.97 | 0.72 | .45 | -0.62 | -1.90 – 0.66 | 0.65 | .34 | |
| Non-Parent | -0.49 | -1.54 – 0.56 | 0.53 | .36 | 0.24 | -0.69 – 1.18 | 0.47 | .61 | |
| Bachelor’s Degree | -0.91 | -1.91 – 0.09 | 0.51 | .07 | 0.08 | -0.78 – 0.94 | 0.44 | .85 | |
| Income | -0.04 | -1.56 – 1.49 | 0.13 | .77 | -0.08 | -0.33 – 0.17 | 0.12 | .52 | |

*Note:* **p*<.05, ***p*<.01, ****p*<.001; Male, Urban, Parent, and No Bachelor’s Degree were used as comparison groups; Similar regressions that used Rural as the comparison group indicated no significant effects of urban status.
