# Supplementary material for: Parental rights or parental wrongs: Parents’ metacognitive knowledge of the factors that influence their school choice decisions
Source: PLoS One. 2024 Apr 18;19(4):e0301768. doi: 10.1371/journal.pone.0301768 (PMC11025896; doi:10.1371/journal.pone.0301768)
Supplement: S1 Fig — (DOCX) [file pone.0301768.s004.docx]

**Study 3 CTR: RAW-AIR, RAW-SAW, and Simulated RAW-SAW Correlations, By Attribute**

*Note.* Error bars reflect 95% confidence intervals. CTR = Control Condition

**Study 3 S1K: RAW-AIR, RAW-SAW, and Simulated RAW-SAW Correlations, By Attribute**

*Note.* Error bars reflect 95% confidence intervals. S1K = Study 1 Attributes, Known Formula Condition

**Study 3 S1UK: RAW-AIR, RAW-SAW, and Simulated RAW-SAW Correlations, By Attribute**

*Note.* Error bars reflect 95% confidence intervals. S1UK = Study 1 Attributes, Unknown Formula condition

**Study 3 S2K: RAW-AIR, RAW-SAW, and Simulated RAW-SAW Correlations, By Attribute**

*Note.* Error bars reflect 95% confidence intervals. S2K = Study 2 Attributes, Known Formula condition.

**Study 3 S2UK: RAW-AIR, RAW-SAW, and Simulated RAW-SAW Correlations, By Attribute**

*Note:* Error bars reflect 95% confidence intervals. S2UK = Study 2 Attributes, Unknown Formula condition.
