# Supplementary material for: Parental rights or parental wrongs: Parents’ metacognitive knowledge of the factors that influence their school choice decisions
Source: PLoS One. 2024 Apr 18;19(4):e0301768. doi: 10.1371/journal.pone.0301768 (PMC11025896; doi:10.1371/journal.pone.0301768)
Supplement: S1 Appendix — (DOCX) [file pone.0301768.s005.docx]

**Attribute Descriptions**

***Note*:** The following are the attribute descriptions that were provided to participants before they completed the Choice-Based Conjoint (CBC) survey. The attributes are listed in alphabetical order, based on their abbreviated names, which appear in parentheses.

**Percent of Seniors Who Take At Least 1 AP Class:** *(AP Enrollment)*

***Included in:*** *Study 1a, Study 1b, Study 3 (Control, Study 1 Attribute conditions)*

This detail indicates the percentage of Seniors (12^th^ graders) who take at least 1 Advanced Placement (AP) Course (a college-level course taken in high school). For each school in this study, the percentage of seniors who take at least 1 AP class will be 20%, 30%, 40%, 50%, or 60%. For reference, the national average is just under 40%.

**Average ACT Score:** *(Average ACT Score)*

***Included in:*** *Study 1a, Study 1b, Study 2, Study 3 (All Conditions)*

This detail indicates the average ACT score (an important test score used by colleges to make enrollment decisions) for students in the school. For each school in this study, the average ACT Score will be 15, 18, 21, 24, or 27. For reference, the national average ACT score is approximately 21, and scores can range from 1 to 36. Please assume that the same percentage of students at each school took the ACT.

**Average Parent Rating of the School:** *(Average Parent Rating)*

***Included in:*** *Study 1a, Study 1b, Study 2, Study 3 (All Conditions*)

This detail indicates the average rating parents of students who go to the school would give the school on a scale of 1 star to 5 stars. Each school in this study will be rated as 1 star, 2 stars, 3 stars, 4 stars, or 5 stars.

**School Crime Rate per 1,000 Students:** *(Crime Rate)*

***Included in:*** *Study 2, Study 3 (Study 2 Attribute conditions)*

This detail indicates the number of crimes (violent and non-violent) that occur in a school each year per 1,000 students. The national average in the United States is about 30 crimes per 1,000 students. For each school in this study, the crime rate per 1,000 students will be 15, 30, 45, 60, or 75.

**State Test Pass-Rate Gap for Disadvantaged Students:** *(Disadvantaged Student Gap)*

***Included in:*** *Study 1a, Study 1b, Study 3 (Control, Study 1 Attribute conditions)*

This detail indicates the difference in the percentage of disadvantaged (i.e., low-income and minority) and non-disadvantaged students in the school who score proficient or higher on state-mandated tests. For example, if the pass-rate gap in a school is 10 percentage points, this indicates that if 50% of non-disadvantaged students score proficient or higher, only 40% of disadvantaged students will do so. For each school in this study, the state test pass rate gap will be 0, 10, 20, 30, or 40 percentage points. For reference, state-level pass-rate gaps typically range from 20-35 percentage points. Please assume that all values given for this detail are an average of math and reading state test scores.

**Emotional Support Score:** *(Emotional Support Score)*

***Included in:*** *Study 2, Study 3 (Study 2 Attribute conditions)*

This detail indicates how well students at a school feel that their school supports them emotionally. The emotional support score is calculated as the average of student ratings on a scale of 1-5 of:

1) How much support the students feel they get at their school;

2) How comfortable the students feel at their school; and

3) How connected the students feel to their teachers.

Emotional support scores can range from 1-5, with 3 being average. Each school in this study will have an Emotional Support Score of 1.2, 2.0, 2.8, 3.6, or 4.4.

**Graduation Rate:** *(Graduation Rate)*

***Included in:*** *Study 1a, Study 1b, Study 2, Study 3 (All Conditions*)

This detail indicates the percentage of students who enter the school as freshmen that graduate after four years. For each school in this study, the graduation rate will be 75%, 80%, 85%, 90% or 95%. For reference, the national average in the United States is around 85%.

**Per-Student Spending:** *(Per-Student Spending)*

***Included in:*** *Study 2, Study 3 (Study 2 Attribute conditions)*

This detail indicates the average amount of money that a school spends per student. The national average is about $12,500, but this number is very different across states because of differences in the cost of living. Please assume that all schools in this study have the same cost of living. For each school in this study, the per-student spending will be $7,000, $9,000, $11,000, $13,000, or $15,000.

**Percent of Students Who Are a Racial/Ethnic Minority:** *(Percent Minority Students)*

***Included in:*** *Study 1a, Study 1b, Study 3 (Control, Study 1 Attribute conditions)*

This detail indicates the percentage of students in the school who are a racial/ethnic minority (i.e., non-white). Each school in this study will have 10%, 30%, 50%, 70%, or 90% racial/ethnic minority students. For reference, the national average is just over 50%.

**Percent of Students Who Pass State Tests:** *(State Test Pass Rate)*

***Included in:*** *Study 1a, Study 1b, Study 2, Study 3 (All Conditions*)

This detail indicates the percentage of students in the school who pass state-mandated tests with a score of proficient or higher. For each school in this study, the percent that pass state tests will be 20%, 35%, 50%, 65%, or 80%. For reference, the national average is between 40-45%, depending on subject. Please assume that all values given for this detail are an average of math and reading state test scores.

**Average Teacher Exam Score Percentile:** *(Teacher Exam Score)*

***Included in:*** *Study 2, Study 3 (Study 2 Attribute conditions)*

This detail indicates how well, on average, the teachers at a school scored on their state licensing exams compared to other teachers. For example, if a school’s Average Teacher Exam Score is in the 90^th^ percentile, this indicates that the teachers at that school, on average, scored better on their licensing exams than 90% of all teachers. This can be thought of as a measure of teacher quality. A score in the 50^th^ percentile is average. For each school in this study, the Average Teacher Exam Score Percentile will be 30^th^, 40^th^, 50^th^, 60^th^, or 70^th^ percentile.

**The Cash Report** (*The Cash Report*)

***Included in:*** *Study 3 (All Experimental Conditions)*

The Cash Report is a new online resource that collects details about schools across the country and uses those details to rate each school on a report card-style A+ to F scale (with A+ being the best and F being the worst). It is similar to other well-known school rating resources such as U.S. News & World Report, Niche, and GreatSchools.com. The Cash Report calculates its ratings primarily based on the following details for each school: the graduation rate, the percent of students who pass state tests, the state test pass-rate gap for disadvantaged students, the average ACT score, the average parent rating of the school, the percent of seniors who take at least 1 AP class, and the percent of students who are a racial/ethnic minority. Each school in this study will be graded as an A-, B, C+, C-, or D. The average Cash Report rating is a C+.

*(Note: The underlined portion is only included in the “Known Formula” conditions.*

**Attribute Levels**

| **Attribute** | **Level 1** | **Level 2** | **Level 3** | **Level 4** | **Level 5** |
| --- | --- | --- | --- | --- | --- |
| AP Enrollment | 20% | 30% | 40% | 50% | 60% |
| Average ACT Score | 15 | 18 | 21 | 24 | 27 |
| Average Parent Rating | 1 Star | 2 Stars | 3 Stars | 4 Stars | 5 Stars |
| Crime Rate (Per 1,000 Students) | 15 | 30 | 45 | 60 | 75 |
| Disadvantaged Student Gap | 0 Percentage Points | 10 Percentage Points | 20 Percentage Points | 30 Percentage Points | 40 Percentage Points |
| Emotional Support Score | 1.2 | 2.0 | 2.8 | 3.6 | 4.4 |
| Graduation Rate | 75% | 80% | 85% | 90% | 95% |
| Percent Minority Students | 10% | 30% | 50% | 70% | 90% |
| Per-Student Spending | $7,000 | $9,000 | $11,000 | $13,000 | $15,000 |
| State Test Pass Rate | 20% | 35% | 50% | 65% | 80% |
| Teacher Exam Score (Percentile) | 30^th^ | 40^th^ | 50^th^ | 60^th^ | 70^th^ |
| The Cash Report | D | C- | C+ | B | A- |
