# Supplementary material for: Parental rights or parental wrongs: Parents’ metacognitive knowledge of the factors that influence their school choice decisions
Source: PLoS One. 2024 Apr 18;19(4):e0301768. doi: 10.1371/journal.pone.0301768 (PMC11025896; doi:10.1371/journal.pone.0301768)
Supplement: S2 Appendix — (DOCX) [file pone.0301768.s006.docx]

**Description of Pilot Studies**

To determine which attributes should be included in Study 2 (and later Study 3), we conducted two pilot studies in which participants were asked to list (Pilot Study 1) and rate the importance of (Pilot Study 2) attributes that parents might evaluate when making school choice decisions.

**Pilot Study 1**

For Pilot Study 1, 100 participants were recruited from Amazon’s Mechanical Turk (MTurk). Participants were asked to imagine that they had a child who was about to start high school, and to list five things (attributes) they would care about when deciding upon which school to send their child. Each participant was then asked to list three things (attributes) that they believed other parents would care about when choosing a high school that the participant did not consider to be important. Participants also completed a brief demographic survey. Before analysis, the data from five participants was removed due to failures to accurately complete a bot check, thus resulting in a total of 95 participants (48 females, 45 males, one non-binary, one missing gender data). Of the 95 participants, 51 (53.68%) had children.

In total, we collected 475 responses regarding attributes that participants believed were important for informing school choice and 285 responses regarding attributes that participants believed *other* parents thought were important for informing school choice. This resulted in a list of 760 total responses. We removed duplicates and non-answers (e.g., “nothing” or “I can’t think of anything”), and the remaining responses were categorized, leading to the identification of 63 unique attributes. These 63 unique attributes were used to inform the second pilot study.

**Pilot Study 2**

In Pilot Study 2, 130 participants were recruited from MTurk. Participants were asked to imagine that they were parents picking a high school to which to send their child and were asked to rate the importance of various attribute statements on a scale of -5 (“it is really important to you that your child does NOT go to a high school for which the statement is true”) to 5 (“it is really important to you that your child DOES go to a high school for which the statement is true”), with responses closer to 0 indicating that the statement was less important to the participant. The participants rated a total of 79 attributes, which consisted of the 63 attributes identified in Pilot Study 1, 14 attributes drawn from school rating websites’ formulas that were not mentioned by participants in Pilot Study 1, and two attributes related to online schooling. Participants also completed a brief demographic survey. Before data was analyzed, 31 responses were removed due to failures to accurately complete a bot check, leaving a total of 99 participants (55 females, 41 males, 1 non-binary, 2 missing gender data). Of these 99 participants, 58 (58.6%) had children.

Across all attributes, average importance ratings ranged from a low of -2.13 (“The school is a religious school”) to a high of 4.54 (“The school is safe”). Most of the attributes (67/79) had a positive average importance rating and the mean of the average importance ratings was 2.25 (*sd* = 1.72). When responses were converted to absolute values at the participant level (i.e., before averaging) to capture importance regardless of valence, average importance ratings ranged from a low of 1.54 (“The school is located in a wealthy area”) to a high of 4.58 (“The school is safe”). The mean of the average importance ratings after the absolute value transformation was 3.04 (*sd* = 0.82). Average importance ratings (both raw and absolute) are reported in the table below.

| Pilot Study 2 Importance Ratings (Raw and Absolute) | | | | |
| --- | --- | --- | --- | --- |
| Attribute (as described to participants) | **Mean Rating** | ***SD*** | **Mean**  **Absolute**  **Rating** | **Absolute**  ***SD*** |
| The school is safe | 4.54 | 1.07 | 4.58 | 0.88 |
| The school is accredited (approved by the state) | 4.48 | 1.12 | 4.51 | 1.03 |
| The school has high academic quality | 4.41 | 0.97 | 4.41 | 0.97 |
| The teachers and staff who work at the school are effective | 4.31 | 1.35 | 4.39 | 1.06 |
| The school is located in a safe area | 4.27 | 1.35 | 4.35 | 1.05 |
| The school has a good reputation | 4.22 | 1.17 | 4.24 | 1.10 |
| The school uses an academically strong curriculum | 4.20 | 1.18 | 4.20 | 1.18 |
| The school's facilities are well-maintained | 4.14 | 1.12 | 4.14 | 1.12 |
| The school has many resources | 4.13 | 1.31 | 4.21 | 1.01 |
| The students at the school are well-prepared for college | 3.95 | 1.73 | 4.13 | 1.23 |
| The school has a high college admissions rate | 3.81 | 1.69 | 3.97 | 1.27 |
| The students at the school do well on the SAT/ACT | 3.78 | 1.77 | 3.96 | 1.31 |
| The students at the school score well on math standardized/state tests | 3.74 | 1.49 | 3.76 | 1.44 |
| The school is highly ranked | 3.73 | 1.57 | 3.79 | 1.42 |
| The students at the school get good grades/have good GPAs | 3.72 | 1.36 | 3.74 | 1.30 |
| The students at the school score well on reading standardized/state tests | 3.70 | 1.73 | 3.84 | 1.38 |
| The school offers strong emotional support for students | 3.68 | 1.35 | 3.68 | 1.35 |
| The students who attend the school think it is a good school | 3.67 | 1.80 | 3.85 | 1.37 |
| The school offers a wide variety of courses | 3.66 | 1.37 | 3.70 | 1.26 |
| The school offers many college-level (e.g., AP or IB) courses | 3.64 | 1.60 | 3.64 | 1.60 |
| The parents of students who attend the school think it is a good school | 3.56 | 1.59 | 3.66 | 1.33 |
| The school has lots of technology | 3.48 | 1.47 | 3.51 | 1.42 |
| The students at the school pass many different college-level (e.g., AP or IB) courses | 3.47 | 1.53 | 3.54 | 1.38 |
| The students at the school take many college-level (e.g., AP or IB) classes | 3.41 | 1.63 | 3.52 | 1.40 |
| Most of the students at the school who take college-level (e.g., AP or IB) courses pass them | 3.41 | 1.56 | 3.45 | 1.47 |
| The school has a high employment rate (post-graduation) | 3.38 | 1.76 | 3.51 | 1.50 |
| The students at the school take many different college-level (e.g., AP or IB) courses | 3.36 | 1.57 | 3.68 | 1.35 |
| The school environment is friendly | 3.23 | 1.80 | 3.33 | 1.60 |
| The school has good gifted and talented education (GATE) services | 3.17 | 1.73 | 3.17 | 1.73 |
| The low-income students at the school perform about as well as other students | 3.13 | 2.06 | 3.39 | 1.59 |
| The school's online options are high-quality | 3.13 | 2.12 | 3.33 | 1.78 |
| The students at the school will be easy for my child to be friends with | 3.10 | 1.39 | 3.10 | 1.39 |
| The students at the school perform better on standardized/state tests each year than the year before | 3.05 | 2.16 | 3.43 | 1.46 |
| The school offers many extracurricular (after-school) opportunities | 2.98 | 1.90 | 3.20 | 1.48 |
| The school is close to home | 2.90 | 1.85 | 3.08 | 1.53 |
| The school offers healthy food options | 2.87 | 1.78 | 2.99 | 1.57 |
| The teachers at the school are rarely absent, so substitutes are not often needed | 2.87 | 1.69 | 2.91 | 1.62 |
| The tuition and fees are low | 2.83 | 2.11 | 4.39 | 1.06 |
| The students at the school do not skip class very often | 2.82 | 2.28 | 3.22 | 1.65 |
| The teachers at the school are well-paid | 2.82 | 1.61 | 2.86 | 1.54 |
| The racial/ethnic-minority students at the school perform about as well as other students | 2.81 | 2.26 | 3.15 | 1.74 |
| The low-income students in the school perform well | 2.71 | 2.34 | 3.17 | 1.65 |
| The class sizes are small | 2.68 | 1.75 | 2.84 | 1.47 |
| The racial/ethnic-minority students at the school perform well | 2.65 | 2.02 | 2.75 | 1.88 |
| The school spends a lot of money per-student | 2.47 | 2.00 | 2.72 | 1.65 |
| The parents of students in the school are very involved in the school | 2.43 | 1.69 | 2.64 | 1.35 |
| The students at the school do better than we would expect based on the demographics of the students that attend the school | 2.40 | 2.39 | 2.89 | 1.76 |
| The students at the school are rarely suspended | 2.38 | 2.16 | 2.71 | 1.73 |
| The staff/faculty are racially/ethnically diverse | 2.13 | 2.53 | 2.72 | 1.88 |
| The student body is racially/ethnically diverse | 2.11 | 2.82 | 3.04 | 1.77 |
| The school has an online option | 2.06 | 2.46 | 2.65 | 1.80 |
| The school offers many food options | 1.88 | 2.10 | 2.32 | 1.59 |
| The school enforces strong discipline | 1.82 | 2.42 | 2.48 | 1.72 |
| The student body is economically diverse | 1.66 | 2.61 | 2.51 | 1.80 |
| The school day starts at a time that is convenient for my family | 1.62 | 2.42 | 2.34 | 1.72 |
| The school's facilities are relatively new | 1.61 | 2.12 | 2.21 | 1.47 |
| The school offers strong special needs accommodations | 1.58 | 2.30 | 2.08 | 1.86 |
| The school offers many arts classes | 1.56 | 2.03 | 1.96 | 1.63 |
| The school offers many different sports programs | 1.55 | 2.30 | 2.17 | 1.72 |
| The school day ends at a time that is convenient for my family | 1.51 | 2.42 | 2.21 | 1.79 |
| The school offers tasty food options | 1.43 | 2.02 | 1.94 | 1.54 |
| The school is a public school | 1.22 | 2.55 | 2.09 | 1.90 |
| The school uses a social-justice focused curriculum | 1.08 | 2.84 | 2.39 | 1.86 |
| The school has been in the neighborhood for a long time | 0.77 | 2.49 | 1.82 | 1.86 |
| The school is located in an area with many fun things to do | 0.68 | 2.42 | 1.89 | 1.65 |
| Most of the students at the school play sports | 0.48 | 2.47 | 1.82 | 1.73 |
| The school spends a lot of money on non-teaching staff | 0.22 | 2.43 | 1.80 | 1.64 |
| The school has many students | -0.09 | 2.41 | 1.81 | 1.58 |
| The school uses a patriotic curriculum | -0.40 | 3.19 | 2.65 | 1.81 |
| The school is located in a wealthy area | -0.65 | 2.24 | 1.54 | 1.75 |
| The school's colors are visually appealing | -0.72 | 2.52 | 1.69 | 1.99 |
| The student body is mostly the same race/ethnicity as my child | -1.05 | 2.91 | 2.40 | 1.94 |
| The school has famous alumni | -1.12 | 2.81 | 2.09 | 2.18 |
| The school is lenient when it comes to discipline | -1.18 | 2.58 | 2.25 | 1.72 |
| The school is the school from which I graduated | -1.19 | 2.96 | 2.34 | 2.16 |
| The school requires uniforms | -1.21 | 2.44 | 2.00 | 1.85 |
| The school has a cool mascot | -1.31 | 2.89 | 2.24 | 2.24 |
| The staff/faculty are mostly the same race/ethnicity as my child | -1.34 | 2.60 | 2.15 | 1.98 |
| The school is a religious school | -2.13 | 3.05 | 3.14 | 1.98 |

*Note:* Attributes selected to be included in Study 2 (and later Study 3) are highlighted in yellow.
