# Supplementary material for: Parental rights or parental wrongs: Parents’ metacognitive knowledge of the factors that influence their school choice decisions
Source: PLoS One. 2024 Apr 18;19(4):e0301768. doi: 10.1371/journal.pone.0301768 (PMC11025896; doi:10.1371/journal.pone.0301768)
Supplement: S1 Text — (DOCX) [file pone.0301768.s007.docx]

**Comparison of Average RAW-SAW Differences Across Study 3 Conditions**

To further evaluate the impact that having access to *The Cash Report* had on participants’ metacognitive knowledge, we compared the mean Average RAW-SAW Differences across the five conditions included in Study 3. A one-way ANOVA suggested significant differences in mean Average RAW-SAW Differences across conditions (*F*(4, 993) = 5.261, *p* < .001, η^2^ = 0.02) and pairwise comparisons using Tukey’s HSDs suggested that average RAW-SAW Differences were significantly higher in the Control condition than in the S1K, S2K, and S2UK conditions (*p*s = <.001 - .009). While this result provides some evidence that access to aggregate school ratings may improve metacognitive knowledge of attribute weights, we are hesitant to place significant weight on this finding because the effect sizes are small and may be driven by the fact that having eight attributes rather than seven reduced the average value of RAWs and SAWs for each attribute, thus artificially deflating mean Average RAW-SAW Differences.

Furthermore, the finding that there were no significant differences between the four experimental conditions (*p*s .21 - .999) indicated that the impact of the *The Cash Report* was not variable based on a) the other attributes with which it was presented; b) participants’ knowledge of how *The Cash Report* was calculated; or c) the redundancy of *The Cash Report.* We argue that this invariance suggests that *The Cash Report* had relatively little influence on metacognitive knowledge of attribute weights, as participants who were using it to augment their metacognitive knowledge likely would have been responsive to changes in how it was presented.
